# A Dexamethasone-Loaded Polymeric Electrospun Construct as a Tubular Cardiovascular Implant

**DOI:** 10.3390/polym15214332

**Published:** 2023-11-06

**Authors:** Stavroula Kyriakou, Sergio Acosta, Ikram El Maachi, Stephan Rütten, Stefan Jockenhoevel

**Affiliations:** 1 Department of Biohybrid & Medical Textiles (BioTex), AME—Institute of Applied Medical Engineering, Helmholtz Institute, RWTH Aachen University, 52074 Aachen, Germany; kyriakou@ame.rwth-aachen.de (S.K.); sacosta@bioforge.uva.es (S.A.); el-maachi@ame.rwth-aachen.de (I.E.M.); 2 Electron Microscopy Facility, University Hospital RWTH Aachen, 52074 Aachen, Germany; sruetten@ukaachen.de; 3 AMIBM—Aachen-Maastricht-Institute for Biobased Materials, Maastricht University, 6167 RD Geleen, The Netherlands

**Keywords:** iPSC-CMs, poly(l-lactide) (PLA), poly(d,l-lactide-*co*-glycolide) (PLGA), fibrin gel

## Abstract

Cardiovascular tissue engineering is providing many solutions to cardiovascular diseases. The complex disease demands necessitating tissue-engineered constructs with enhanced functionality. In this study, we are presenting the production of a dexamethasone (DEX)-loaded electrospun tubular polymeric poly(l-lactide) (PLA) or poly(d,l-lactide-*co*-glycolide) (PLGA) construct which contains iPSC-CMs (induced pluripotent stem cell cardiomyocytes), HUVSMCs (human umbilical vein smooth muscle cells), and HUVECs (human umbilical vein endothelial cells) embedded in fibrin gel. The electrospun tube diameter was calculated, as well as the DEX release for 50 days for 2 different DEX concentrations. Furthermore, we investigated the influence of the polymer composition and concentration on the function of the fibrin gels by imaging and quantification of CD31, alpha-smooth muscle actin (αSMA), collagen I (col I), sarcomeric alpha actinin (SAA), and Connexin 43 (Cx43). We evaluated the cytotoxicity and cell proliferation of HUVECs and HUVSMCs cultivated in PLA and PLGA polymeric sheets. The immunohistochemistry results showed efficient iPSC-CM marker expression, while the HUVEC toxicity was higher than the respective HUVSMC value. In total, our study emphasizes the combination of fibrin gel and electrospinning in a functionalized construct, which includes three cell types and provides useful insights of the DEX release and cytotoxicity in a tissue engineering perspective.

## 1. Introduction

Cardiovascular diseases constitute a significant burden worldwide [1]. In response to this pervasive health issue, tissue engineering has emerged as a promising avenue, offering numerous solutions for treating various cardiovascular diseases. These solutions encompass a wide spectrum, ranging from arterial replacements, vascular conduits, perivascular implants, heart valves, and myocardium and cardiovascular implants [2]. Yet, within this intricate landscape of cardiovascular health, a distinct pathological condition prevails—atrioventricular block, primarily attributed to idiopathic fibrosis of the conduction system. Remarkably, this condition can manifest without the presence of overt heart disease [3]. Management of atrioventricular block often necessitates pacemaker implantation, depending on the degree of signal delay [4,5]. However, while these electronic pacemakers have significantly advanced the field of cardiology, they are not without their limitations, particularly when implanted in pediatric patients, where the adverse impact on quality of life becomes especially pronounced [6,7,8].

In recent years, there has been a growing interest in the development of biologic pacemakers as an alternative to traditional electronic pacemakers. This paradigm shift seeks to leverage the potential of induced pluripotent stem cell-derived cardiomyocytes (iPSC-CMs) in the creation of self-regulating cardiac pacemakers. In this context, numerous approaches involving induced pluripotent stem cell-derived cardiomyocytes (iPSC-CMs) have been explored. Due to the drawbacks associated with pacemaker implantation in pediatric patients, many studies have investigated cell-based approaches to restore the electrical signal. For instance, the tissue maturity of iPSC-CMs was tested in a platform providing exogenous biophysical stimuli [9] and many more studies have investigated the three-dimensional heart tissues [10]. Chaveau et al. proved that iPSC-CMs induce in vivo pacemaker function [11], while Zhang et al. created engineered conductive tissue derived from cardiac progenitor cells [12]. However, for the evaluation of the tissue-engineered heart tissues, there are different systems needed [13].

Moreover, various cutting-edge technologies have been harnessed to produce scaffolds tailored for cardiac tissue engineering. These encompass microfluidics [14], 3D printing [15] and organoids [16]. Notably, electrospinning has emerged as a crucial biofabrication method, offering the versatility to create fibers of varying dimensions [17,18,19]. Many polymers have been processed via the electrospinning method to be used as scaffolds for cell seeding afterwards. For instance, Johanne et al. cultivated human cardiomyocytes on collagen scaffolds to induce cardiac remodeling in delated cardiomyopathy [20]. Moreover, Li et al. seeded human iPSC-CMs on PMGI fibers through extracellular recording [21]. Furthermore, PCL was used to investigate the influence of anisotropic and oriented scaffolds on iPSC-CMs [22,23], while Chun et al. combined PCL with polyethylene glycol and carboxylated polycaprolactone to facilitate in vitro maturation of iPSC-CMs [24].

Beyond the application of polymeric scaffolds alone, studies have delved into the combination of polymers with glucocorticoid steroids for enhancement cell treatment [25,26]. Dexamethasone (DEX), a glucocorticoid steroid with a rich therapeutic history spanning the treatment of multiple sclerosis, allergies, cerebral edema, inflammation, and shock [27], has shown promise in promoting robust T-tubule development and enhancing extracellular cardiomyocyte coupling within scaffolds [28,29]. However, it is noteworthy that glucocorticoids have been associated with increased cytotoxicity levels in vascular endothelial cells [30].

In this study, we are fabricating polymeric tubular constructs made of PLA and PLGA including DEX using the electrospinning method and evaluating the release profiles of DEX from these constructs. Additionally, we describe the methodology employed to embed three distinct cell types within fibrin gel molds housed within the polymeric tubular constructs. Our investigation extends to assess cell viability by monitoring the expression of critical extracellular matrix proteins. Furthermore, we undertake an evaluation of cytotoxicity and cell proliferation concerning human umbilical vein endothelial cells (HUVECs) and human umbilical vein smooth muscle cells (HUVSMCs) on polymeric sheets. The overarching goal of this study is to develop a polymeric construct capable of retaining iPSC-CM, HUVEC, and HUVSMC functionality while enabling the controlled release of DEX. Overall, we aim to clarify some critical parameters in the development of biologic pacemakers as innovative solutions for cardiac rhythm management.

## 2. Materials and Methods

Poly(l-lactide) (PLA) (Sigma Aldrich, Saint Louis, MO, USA, MW 85,000–160,000) and Poly(d,l-lactide-*co*-glycolide) (PLGA) (Sigma-Aldrich Saint Louis, MO, USA, lactide: glycolide 75:25, mol wt 66,000–107,000) were dissolved in a solvent system containing 2,2,2-Trifluorethanol (Carl Roth, Karlsruhe, Germany, >99.8%) and chloroform (Sigma Aldrich, Saint Louis, MO, USA, ≥99.8%) in a 4:1 ratio. The polymer concentration was 150 mg/mL. The solutions were magnetically stirred at room temperature for 8 h at 500 rpm and used within 2 days. DEX (Sigma-Aldrich Saint Louis, MO, USA, BioReagent, ≥97%) was added in the polymer powder (added glass bottles before adding the solvent) for the preparation of solutions containing 20 mg/mL and 40 mg/mL of DEX. Table 1 lists an overview of the samples prepared and their abbreviations.

### 2.1. Fabrication of the Fibrillary Scaffolds by Electrospinning

**Figure 1:**
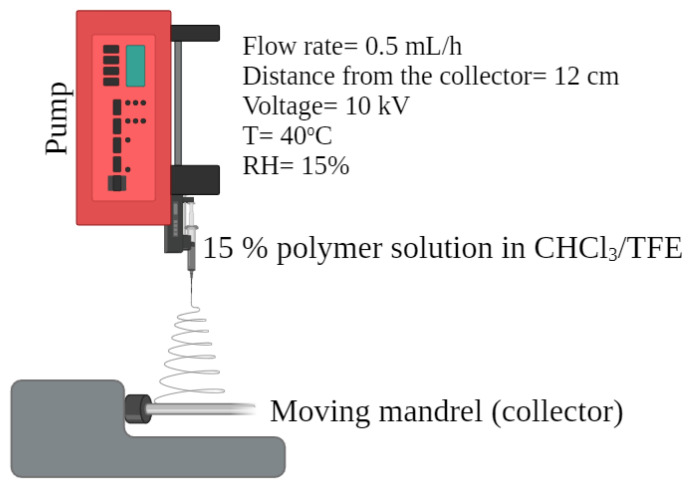
Schematic representation of the electrospinning process.

The electrospinning method, as described by Doshi and Reneker [31], was applied using a syringe pump (Igus, Cologne, Germany). The collector was either a metal rod (for the tubular structures) or aluminum foil (for the flat structures) and the distance from the needle was fixed at 12 cm (Figure 1). The pump flow rate was 0.5 mL/h with an applied voltage of 10 kV in the polymer solution. A negative voltage with a value of 0.5 kV was applied to the collector. The temperature was maintained at 40 °C and the humidity at 15%. Each electrospinning process ran for 30 min and the constructs were incubated under the same temperature and humidity conditions overnight to achieve efficient solvent evaporation.

### 2.2. Scanning Electron Microscopy

Scanning electron microscopy (SEM) was used to evaluate the fibrillar microstructure of the different configurations for the tubular constructs. The samples were sputter-coated with 12.5 nm Pd-Au (EM SCD500, Leica, Wetzlar, Germany). To observe the interior of the fibers, samples were glued to carbon tape and carefully fractured, splitting the fiber and exposing the interior. Samples were analyzed in high-vacuum mode using an ESEM XL 30 FEG (FEI, Eindhoven, Netherlands) at an acceleration voltage of 10 kV.

### 2.3. Evaluation of the Electrospun Fiber Diameter

For the diameter measurement, random SEM micrographs from three different samples were analyzed. The sample images in magnification 5000× were used for each polymer concentration. The Diameter Jplug-in (software version 1.018) from the Image J (software version 1.51 w) [32] was used for the image processing. The best segmentation image was selected, and the diameter was expressed as the mean fiber diameter from the triplicates selected.

### 2.4. Drug Release Study

The tubular constructs were removed from the metal rods and placed in 0.9 mm diameter cannulas. As for the sheets, they were cut into 1 cm^2^ pieces and used for the cytocompatibility tests. The constructs were sanitized using UV irradiation for 1 h at room temperature. After the sanitation, the 20 mg/mL and 40 mg/mL DEX containing polymeric cylindrical samples were added to Eppendorf vials (Thermo Fisher, Waltham, MA, USA), immersed in 1 mL PBS, and incubated in a platform shaker at 37 °C for 50 days (Titramax 1000, Heidolph Instruments, Schwabach, Germany). For each sample measurement, 2 µL of the solution was used and the concentration of the drug in the solutions was measured with a Nanodrop One C device (Thermo Fisher, Waltham, MA, USA) at 242 nm. The mass of the released DEX was calculated using a standard curve based on Equation (1) (Figure S1), where *y* is the calculated mass of dexamethasone in µg/mL and *x* is the absorption measured at 242 nm. The data were expressed as a percentage of DEX release based on the initial amount of dexamethasone added to the polymer solution.

(1) y=47.071×x+0.5448

Equation (1)—Calibration curve for dexamethasone.

### 2.5. Hydrogel in the Polymer Tubular Structures

**Figure 2:**
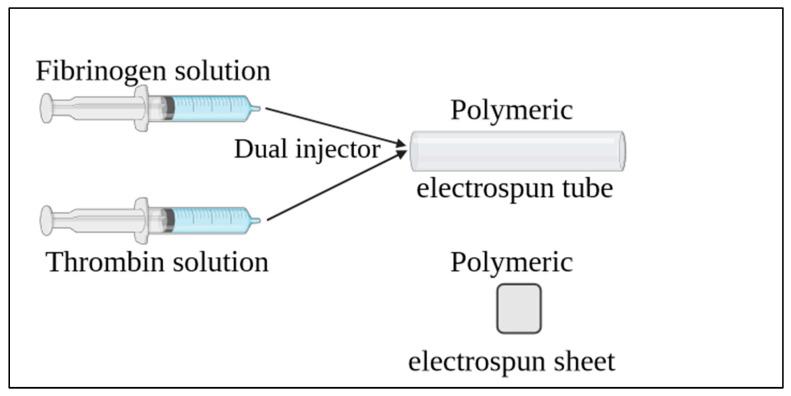
Electrospun constructs and molding of the fibrin gels.

Fibrin gels containing iPSC-CMs, HUVSMCs, and HUVECs, or only HUVSMCs or HUVECs were prepared. The concentration of the cell suspension was 1 million/mL, 1 million/mL, and 15 million/mL for HUVSMCs, HUVECs, and iPSC-CMs, respectively. Briefly, the solutions for the fibrin polymerization were prepared as described by Keijdener, et al. [33]. The molding procedure took place either in the polymeric tubular constructs or on top of the polymeric sheets (Figure 2). The iPSC-CMs containing gels were cultivated with a medium consisting of 25% DMEM (Thermo Fisher, Waltham, MA, USA), 25% EGM-2 (PromoCell, Heidelberg, Germany) and 50% Plyricyte medium (Ncardia, Leiden, The Netherlands) supplemented with Aprotinin (1000 U/mL, Nordic Group, Singapore) and antibiotic medium (ABM, Pan Biotech, Aidenbach, Germany). The other 2 kinds of samples were cultivated using ABM and aprotinin only with DMEM for the HUVSMCs and only with EGM-2 for the HUVECs. The medium was changed at day 2, 4, 6, 8, 10, 12, and 14. For the samples containing iPSC-CMs, cylindrical fibrin gels were prepared as previously described [34], and were inserted inside the polymeric tubular structures on day 3, 7, and 10 of the cultivation period.

### 2.6. Cell Culturing

Human umbilical vein smooth muscle cells (HUVSMCs) and human umbilical vein endothelial cells (HUVECs) were handled as described previously [35]. After written consent, The University Hospital Aachen, Aachen, Germany provided the umbilical cords by the RWTH Aachen University Centralized Biomaterial Bank (cBMB) according to its regulations, following the RWTH Aachen University, Medical Faculty Ethics Committee’s approval (cBMB project number 323). For the HUVSMCs, Dulbecco’s Modified Eagle’s Medium (DMEM, Thermofischer, Waltham, MA, USA) supplemented with 10% FCS was used, while for HUVECs, EGM (PromoCell, Heidelberg, Germany) was supplemented with 1% FCS, basic Fibroblast Growth Factor, Insulin-like Growth Factor, Vascular Endothelial Growth Factor 165, Ascorbic Acid, Heparin, and Hydrocortisone. Both HUVSMCs and HUVECs were up to passage 5. Induced pluripotent stem cell cardiomyocytes (iPSC-CMs) and their culture medium (Plyricyte) were purchased by Ncardia, (Leiden, the Netherlands). The iPSC-CMs were plated using 10 μg/mL fibronectin (Sigma-Aldrich, Saint Louis, MO, USA), while for the HUVECs, 2% gelatin (Sigma-Aldrich, Saint Louis, MO, USA) was used. The HUVSMCs and HUVECs were cultured at 37 °C, 5% CO_2_; the medium was changed every three days and they were up to passage 5. Trypsin (Thermo Fisher, Waltham, MA, USA) was used to dissociate the HUVSMCs and HUVECs, while TrypLE Select Enzym (1×, Thermofischer, Waltham, MA, USA) was used for the iPSC-CMs.

### 2.7. Immunohistochemistry

For sample fixation, 4% PFA solution (Carl-Roth, Karlsruhe, Germany) was used at room temperature for 1 h. Afterwards, the samples were washed twice with PBS (Thermofischer, Waltham, MA, USA) and stored at 4 °C until the staining process. For cell membrane permeabilization, 5% normal goat serum and 0.1% Triton X-100 (Sigma-Aldrich, Saint Louis, MO, USA) in PBS were used. The primary antibody incubation took place overnight at 37 °C. Subsequently, the samples were washed two times with PBS and incubated for 8 h at 37 °C. The samples were then incubated with the secondary antibody. After the last washing step, the nuclei were stained with DAPI, incubated for 15 min at 37 °C, and washed three times. The samples were stored at 4 °C in PBS containing 1% ABM until visualization. Table 2 lists the primary and secondary antibodies used. Samples were embedded in 2% agarose gel in PBS and visualized by two-photon laser-scanning microscopy (TPLSM) using an Olympus FluoView 1000MPE with a 25× water objective (NA 1.05, Olympus, Tokyo, Japan), a mode-locked MaiTai DeepSee Titanium-Sapphire Laser (Spectra-Physics, Stahnsdorf, Germany), and FluoView FV 10 4.2 acquisition software.

### 2.8. Beating Frequency

An Okolab heating box (Ottaviani, NA, Italy) was used to incubate the constructs at 37 °C and 5% CO_2_ which were visualized with a Nikon Ti-Eclipse epifluorescence microscope TI-S-CON (Tokyo, Japan). For each sample, a video with duration 1 min was recorded and the beating frequency per minute was calculated considering the iPSC-CM spots beating simultaneously.

### 2.9. Quantification of the Markers

The images were evaluated using Imaris Software (version 9.3). The pixels were counted using the masking option in the surfaces menu for the three different colors (blue, red, green) and the whole set of volume was used for the quantification of the different markers.

### 2.10. Cell Proliferation

The proliferation of HUVECs and HUVSMCs was evaluated by using a Cell Counting Kit 8 (WST-8/CCK8) (ab228554, Abcam, UK). The samples used were either fibrin gels molded on top of the polymeric sheets or polymeric sheets embedded in the respective cell suspension in a concentration of 10,000 cells/cm^2^. The results were expressed as number of living cells, measuring the absorbance at 450 nm. The assay was performed on day 1, 3, and 7 of the cultivation.

### 2.11. Cytotoxicity Evaluation

The cytotoxicity of the polymeric constructs was evaluated through the Lactate Dehydrogenase (LDH) Assay Kit (ab197000, Abcam, UK). The polymeric squares were placed in 48-well plates and the cell suspension (HUVSMCs or HUVECs) was added on them with a concentration of 100,000 cells/mL. The cells were treated with 0.1% Triton X-100 for the positive control, while the negative control was composed only of cells cultured on gelatin. The lactate dehydrogenase activity was measured in the samples and the cytotoxicity was expressed as relative fluorescence units (ΔRFU).

### 2.12. Statistics

All data were represented as mean-SD from three experiments. For the DEX release, the beating frequency, the LDH activity, and the diameter measurements, one-way analysis of variance (ANOVA) was performed using Graph Pad Prism (version 8.4.2). For the quantification of the TPLSM results and the cell proliferation, Tukey´s multiple comparisons test with two-way analysis of variance (ANOVA) was selected. A *p*-value < 0.05 was considered statistically significant.

## 3. Results

### 3.1. Fiber Diameter Measurement

**Figure 3:**
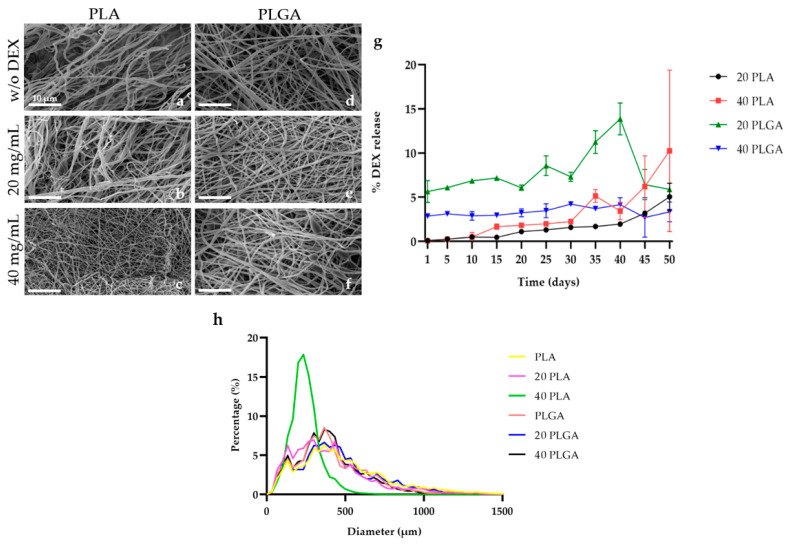
(**a**–**f**) Representative SEM micrographs of the electrospun scaffolds manufactured from PLA and PLGA solutions loaded with varying DEX concentration. (**g**) Percentage of DEX release. 20 PLA (black), 40 PLA (red), 20 PLGA (green), and 40 PLGA (blue). (**h**) Average frequency diameter chart for the PLA, 20 PLA, 40 PLA, PLGA, 20 PLGA, and 40 PLGA constructs.

Figure 3a–f present the SEM micrographs of the PLA and PLGA constructs with varying concentrations of DEX. Supplementary Figure S1 presents the SEM micrographs used for the diameter measurement (Figure S2a–f) and the representative tubular structure of the constructs, too (Figure S2g–i). The PLA fibrillary constructs exhibited the fibers with the highest diameter, closely followed by the 20 PLGA constructs, while the 40 PLA constructs showed the fibers with the smallest diameter. Looking more deeply into each polymer, the fiber diameter difference between the PLA and the 40 PLA was 40%. Therefore, by increasing the DEX concentration, the fiber diameter was decreased (Figure 3h). On the contrary, for the PLGA constructs, the diameter of the 20 PLGA and 40 PLGA constructs was higher than the PLGA ones, and simultaneously, the highest fiber diameter value was observed for the 20 PLGA constructs. Because of this, the average diameter of the PLGA fibers was higher than the PLA fibers (Figure S3b). At the same time, the lowest diameter was observed for the 40 PLA sample (Figure S3a). Consequently, the tendency of the change in the fiber diameter was different for the PLA and PLGA constructs with the various DEX concentrations. However, there were no significant differences among the different polymers and DEX concentrations.

### 3.2. In Vitro Evaluation of the Dexamethasone (DEX) Release

The percentage of DEX released was assessed in both PLA and PLGA electrospun scaffolds (Figure 3g). All the constructs presented an increasing trend in DEX release until day 20 of cultivation. On day 20, the 20 PLGA sample exhibited a temporary decrease in the release, followed by a continuous increase over the next 10 days. Nevertheless, its final release level was similar to that of the 20 PLA sample. Conversely, the 40 PLA sample exhibited a steady increase in DEX release, reaching 10% by the end of the study. Notably, the percentage of DEX release was directly proportional to the DEX concentration in the PLA samples. In contrast, the 40 PLGA sample exhibited a higher DEX release compared to the PLA samples, but the 20 PLGA sample initially had a higher release percentage, which it maintained throughout the study.

### 3.3. Evaluation of the Cytocompatibility of the Electrospun Scaffolds

**Figure 4:**
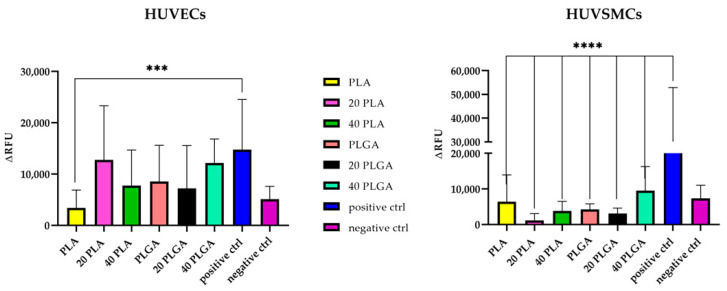
LDH activity of the HUVEC and HUVSMC samples. PLA (yellow), 20 PLA (pink), 40 PLA (green), PLGA (orange), 20 PLGA (black), 40 PLGA (aquamarine), positive control (blue), and negative control (purple). Data are expressed as mean ± SD, *n* = 3, *** *p* < 0.001, **** *p* < 0.0001).

The cytocompatibility of electrospun scaffolds was assessed with two primary cell types relevant to cardiovascular applications, namely, HUVECs and HUVSMCs. This assessment was performed by measuring LDH release after direct contact with the different scaffolds. The assays involving HUVECs indicated that scaffolds made from PLGA exhibited higher cytotoxicity compared to those made from PLA (Figure 4). The toxicity of the polymeric LDH activity was evaluated for the polymer samples, with one positive and one negative control used for both HUVECs and HUVSMCs. For the HUVECs, the PLA sample exhibited the highest cytocompatibility, while the remaining samples were considered cytotoxic, as the absorbance was higher than the negative control. For the HUVSMCs, none of the samples showed significant differences in terms of LDH release with respect to the negative control (cells cultured on gelatin).

### 3.4. Cell Proliferation

**Figure 5:**
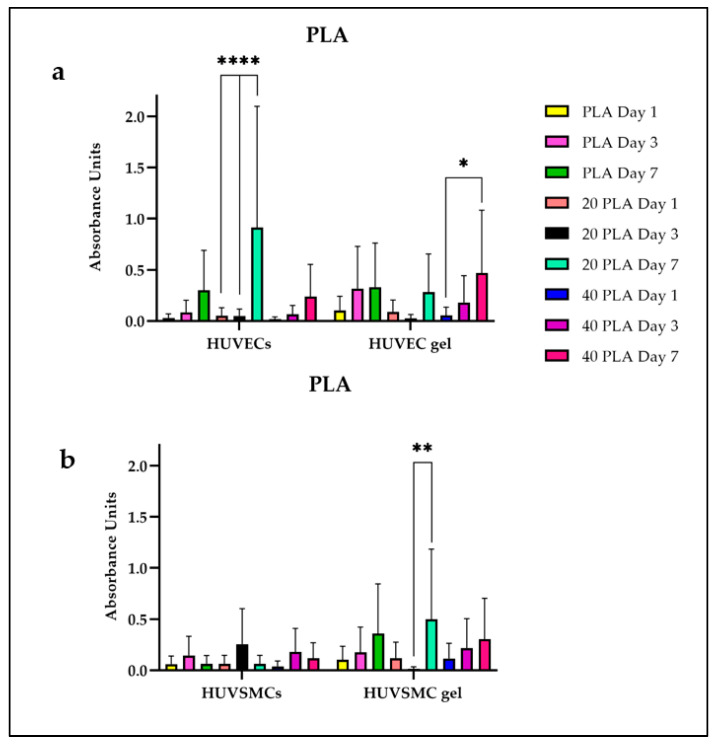
(**a**) Cell proliferation of the HUVECs andHUVEC gel for the PLA samples. (**b**) Cell proliferation for the HUVSMCs and HUVSCM gel for the PLA samples. Data are expressed as mean ± SD, *n* = 3, * *p* < 0.05, ** *p* < 0.01, **** *p* < 0.0001).

**Figure 6:**
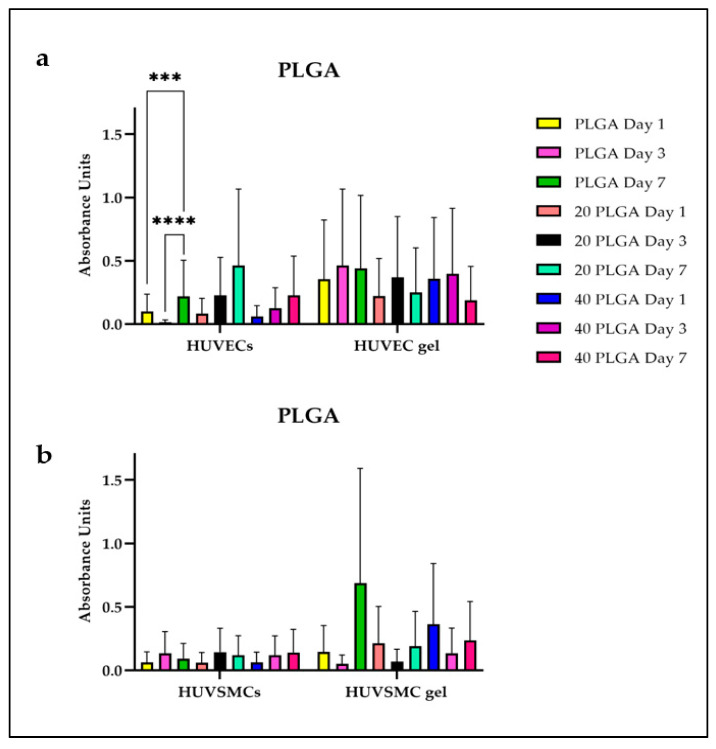
(**a**) Cell proliferation of the HUVECs and HUVEC gel for the PLA samples. (**b**) Cell proliferation for the HUVSMCs and HUVSCM gel for the PLGA samples Data are expressed as mean ± SD, *n* = 3, *** *p* < 0.001, **** *p* < 0.0001).

The cell proliferation on the polymer constructs was evaluated using the Cell Counting Kit 8 and expressed absorbance units for the PLA and the PLGA constructs. For each polymer scaffold, the test was performed by incubating the constructs together with cells and together with cell-embedded fibrin gels, thus mimicking the final composite composition of a biological pacemaker. As Figure 5a shows, the highest proliferation rate for the PLA constructs was observed for the HUVEC 20 PLA sample on day 7 with the value being significantly different from the ones on day 1 and day 3. In parallel, the HUVEC gel 40 PLA sample on day 7 had a proliferation of 0.5% with the value being significantly higher than the value of the 40 PLA sample on day 1. As for the HUVSMC gel, there was a significant difference in the cell proliferation between the 20 PLA day 7 sample and the 20 PLA day 3 sample, even if the 20 PLA day 1 value is higher than the respective one on day 3 (Figure 5b). Moreover, there were no significant differences among the HUVSMC samples. Concerning the cell proliferation on the PLGA samples (Figure 6), the highest value was presented for the HUVSMC gel 20 PLGA day 7 sample being similar with the respective value for the HUVEC gel. However, the only significant differences were shown for the HUVEC samples and especially for the PLGA sample where the value for day 7 is significantly higher than the respective ones of day 1 and day 3. Particularly, the same tendency was observed, as for the previously mentioned 20 PLA samples in the HUVSMC gel (Figure 6b).

We also evaluated the cell proliferation for day 1, day 3, and day 7 separately (Figure S4). Focusing on day 7 for the HUVEC samples, we observed that the 20 PLA sample has the higher proliferation rate among the HUVEC samples, while the value presented a high significant difference among the PLA and 40 PLA samples and a low significant difference compared to the 20 PLGA sample. Observing this relatively high proliferation (approaching 1%), it was higher than the HUVEC gel value and the respective HUVSMC sample value, where the proliferation observed was among the lowest of all the four sets of samples. For the HUVSMC gel, the 40 PLGA sample showed the highest proliferation unlike the 40 PLGA and the 20 PLGA samples, with the lastly mentioned one presenting the lowest cell proliferation among the HUVSMC set of samples. Even if the PLGA HUVSMC sample presented the second high after the 20 PLA sample among the four sets of samples, the HUVSMC PLGA value was among the lowest proliferation values and therefore, significantly different than the HUVSMC gel PLGA value.

### 3.5. CD31, αSMA, Col I as Markers of the HUVECs and HUVSMCs

To investigate the effect of DEX on cell behavior, we evaluated the expression of CD31 in polymeric constructs embedded with HUVECs and the expression of αSMA and col I for the HUVSMCs containing polymeric constructs. Figure S5 shows the CD31 expression for the three different concentrations of PLA on days 4, 8, and 12 of cultivation. After 4 and 8 days of cultivation, the CD31 expression of HUVECs seeded on the PLA and 20 PLGA scaffolds maintained a similar profile. Although on day 12 the CD31 expression presented a cobblestone structure, the respective performance of the 20 PLA sample was not as sufficient as on the previous cultivation days. As for the sample 40 PLA, even if the number of nuclei remained almost the same during the culture, the CD31 signal was stronger on day 12 of the cultivation. Concerning the PLGA constructs and especially on day 4, the CD31 was similarly expressed for all the different polymer concentrations (Figure S6). However, on day 8 and day 12 of the cultivation, the DAPI signal was not as strong as on day 4, but at the same time, the CD31 expression was increased.

For a deeper investigation of the CD31 expression on the two different tubular scaffolds, we quantified the CD31 expression during the cultivation period. Firstly, the number of nuclei is highly significantly different between day 4 and day 8 for the PLA sample while for the 20 PLA sample, the significant difference is observed for day 8 and in combination with day 4 and day 12 (Figure S7a). Unlike the increase in the DAPI signal during the cultivation period for the PLA sample, the CD31 expression was decreased and was highly significantly different from the initial CD31 expression on day 4. However, the CD31 expression of the 20 PLA sample on day 8 was increased since the beginning of the culture, on the contrary to the respective DAPI signal and the CD31 percentage values which were highly significantly different than the CD31 percentage on day 4 and on day 12. Continuing with the PLGA samples (Figure S7b), there was a highly significant difference between the PLGA on day 4 and the PLGA on day 12 samples. A similar tendency was observed for the 20 PLGA samples, but reversibly, as the DAPI signal was decreasing from day 4 to day 12 of the cultivation. The same decreasing tendency was observed for the 40 PLGA samples between day 4 and day 8, but with lower significance importance. As for the CD31 expression and the PLGA samples, there were observed highly significant changes in the CD31 percentage as the expression percentage increased at first on day 8 and then followed a decreasing profile (day 12) for the 20 PLGA samples. However, for the 40 PLGA, the CD31 levels were increased without presenting any significant differences.

**Figure 7:**
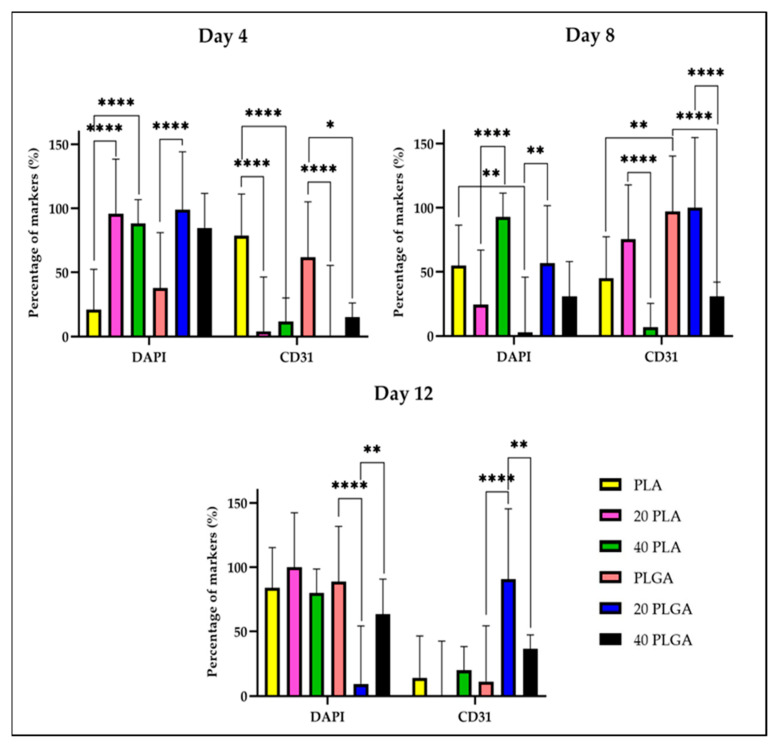
Quantification of DAPI and CD31 on day 4 (**top left**), day 8 (**top right**), and day 12 (**bottom**). Data are expressed as mean ± SD, *n* = 3, * *p* < 0.05, ** *p* < 0.01, **** *p* < 0.0001).

Comparing the different polymeric materials during the cultivation period, we concluded that the CD31 expression on day 4 of the cultivation for the PLA and PLGA samples was higher than the 40 PLA and 40 PLGA samples, respectively (Figure 7). Moreover, the first mentioned values were significantly higher than the 20 PLA and 20 PLGA samples, respectively. On day 8, the CD31 expression of the PLGA sample is significantly higher than the respective PLA samples, while the CD31 expression of the 40 PLA sample is significantly lower than the value of the 20 PLA sample. As for the PLGA samples on day 12, the CD31 percentage was significantly lower than the value of the 20 PLGA sample on day 12 which was significantly higher than the CD31 expression of the 40 PLGA on day 8 and day 12 of the cultivation period.

Furthermore, the expression of αSMA and col I was investigated for their polymeric constructs, which were molded with HUVSMC fibrin gels. As Figure S8 shows, there was sufficient expression of all the markers in all the sets of the samples. Moreover, the αSMA signal was higher for the 20 PLA sample on day 8 and day 12. In Figure S9, the signal of αSMA and col I was not as proportional as the respective pictures in Figure S8. For the PLGA sample, there was not sufficient αSMA expression until the end of the cultivation period, but the col I expression was obvious only on day 12. Concerning the 20 PLGA sample, the col I expression was more obvious on day 8 of the cultivation and the signal intensity remained similar until day 12 of the cultivation. For the 40 PLGA sample, we could observe the αSMA expression on day 4 of the cultivation, but the col I expression was only visible on day 12.

**Figure 8:**
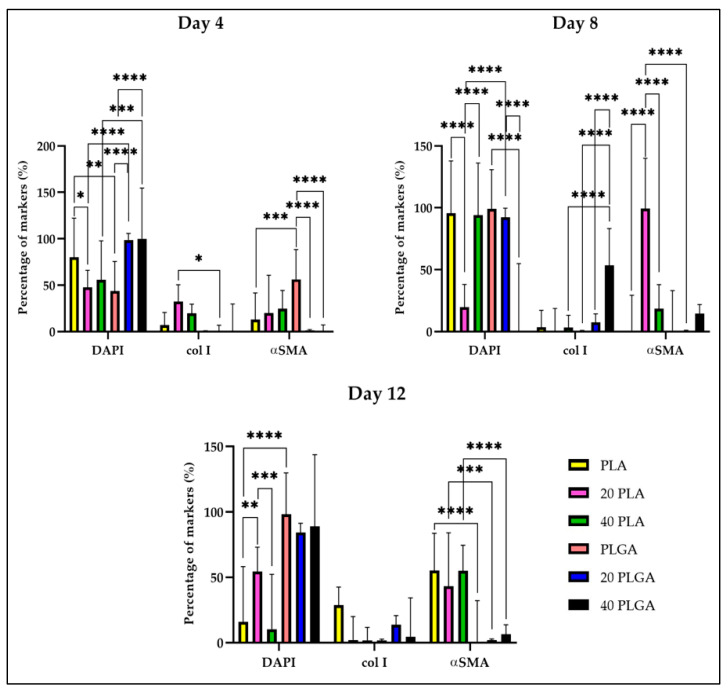
Quantification of DAPI, col I, and αSMA for the PLA and PLGA samples on day 4 (**top left**), day 8 (**top right**), and day 12 (**bottom**). Data are expressed as mean ± SD, *n* = 3, * *p* < 0.05, ** *p* < 0.01, *** *p* < 0.001, **** *p* < 0.0001).

For a more thorough investigation of the expression of the col I and αSMA, we observed that there were highly significant differences among the αSMA expression of the samples (Figure S10), although on day 12, the percentage of αSMA retained similar levels. Overall, for the PLGA samples there was efficient αSMA production only for the 40 PLGA samples on day 12 of the cultivation. Simultaneously, the expression of col I that was the highest among the samples was the one of the 40 PLGA sample on day 8 (Figure 8). Nevertheless, the col I expression value for the 40 PLGA sample on day 12 was lower than the respective value of the day 8 sample with high significance.

Especially on day 4, the col I expression was significantly higher for the 20 PLA sample than the one for the 20 PLGA sample. At the same time, the percentage of the αSMA was increasing as the DEX concentration in the samples was increasing. Nonetheless, the expression of αSMA was the highest for the PLGA sample among the PLGA samples on day 4, but was almost absent for the 20 PLGA and 40 PLGA samples. For day 8, the col I had the highest percentage for the 40 PLGA sample and was highly significantly different from the respective values of the PLGA and the 20 PLGA samples. As for the αSMA, the highest percentage was observed for the 20 PLA sample and was highly significantly different from the PLA, 40 PLA, and 20 PLGA samples. Moreover, on day 12, the highest col I percentage was observed for the PLA sample, although there were no significant changes observed. Additionally, the PLA sample presented the best performance on day 12, as the expression of the αSMA (together with the 40 PLA sample) was the highest among the samples. In parallel, the last value was highly significantly different from the PLGA sample and the same trend was followed comparing the PLA and PLGA samples with the same DEX concentration.

### 3.6. SAA, Cx43, CD31 as Markers of the iPSC-CMs Containing Constructs

For the investigation of the iPSC-CM maturity and electrical conductivity in the electrospun scaffolds, the constructs were stained for SAA and Cx43. Additionally, the CD31 was the marker selected to be evaluated for the HUVECs included in the iPSC-CM constructs.

**Figure 9:**
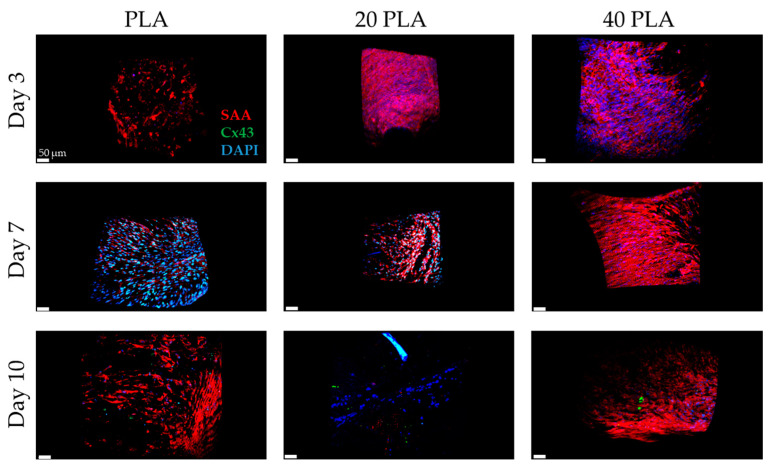
DAPI (blue), αSMA (red), and Cx43 (green) staining for the PLA (**left**), 20 PLA (**middle**), and 40 PLA samples (**right**) on day 3 (first row), day 7 (second row), and day 10 (third row). Scale bar 50 μm.

Figure 9 presents the SAA and the Cx43 levels showing a sufficient amount for day 3 of the cultivation, which was increased until day 10 of the cultivation period. However, for the 20 PLA sample, day 10 does not follow the same tendency as the PLA and the 40 PLA samples. As for the 40 PLA sample, there was sufficient expression of the SAA throughout the cultivation period. As for the Cx43 signal, it was observed stronger on day 7 unlike day 3 and day 10.

**Figure 10:**
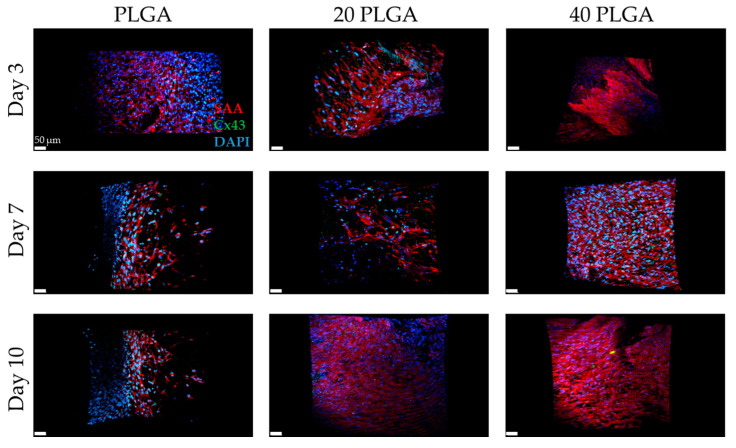
DAPI (blue), αSMA (red), and Cx43 (green) staining for the PLGA (**left**), 20 PLGA (**middle**), and 40 PLGA samples (**right**) on day 3 (first row), day 7 (second row), and day 10 (third row). Scale bar 50 μm.

Concerning the PLGA samples, there was a sufficient amount of SAA expression and the DEX seemed to enhance the expression of SAA with the exemption of the 20 PLGA sample on day 10 (Figure 10). In parallel, the Cx43 signal appeared to be lower for the samples containing DEX.

**Figure 11:**
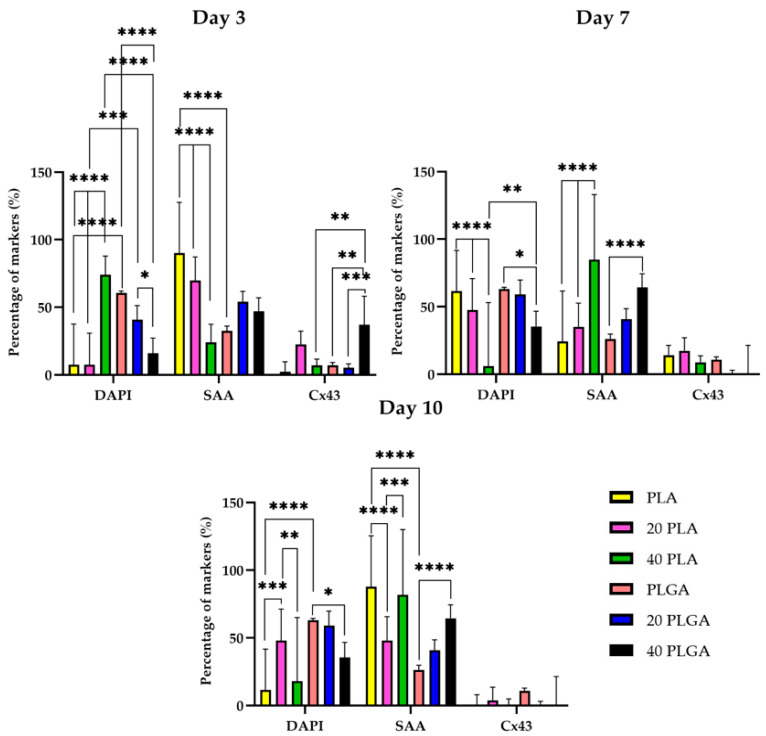
Quantification of DAPI, SAA, and Cx43 for the PLA and PLGA samples on day 3 (**top left**), day 7 (**top right**), and day 10 (**bottom**). Data are expressed as mean ± SD, *n* = 3, * *p* < 0.05, ** *p* < 0.01, *** *p* < 0.001, **** *p* < 0.0001).

The spatiotemporal quantification of SAA revealed that the iPSC-CMs on day 3 with the PLA sample had the strongest signal among the rest of the samples with a highly significant difference from the 20 PLA, 40 PLA, and the PLGA sample (Figure 11). Simultaneously, the highest Cx43 expression was presented for the 40 PLGA sample and was significantly higher than the other PLGA samples. At a later timepoint on day 7, the SAA expression of the PLA sample was decreased and the value was significantly different than the values of the 20 PLA and 40 PLA samples. Moreover, the SAA percentage was slightly increased on day 7 and the same pattern was followed on day 10 of the cultivation. On day 10, the only sample that retained the high SAA expression was the 40 PLA and the value was significantly higher than the value of the 20 PLA sample. As for the Cx43 expression, the DEX-containing PLGA samples presented a very weak signal on day 7 and on day 10 as well.

### 3.7. Beating Frequency

**Figure 12:**
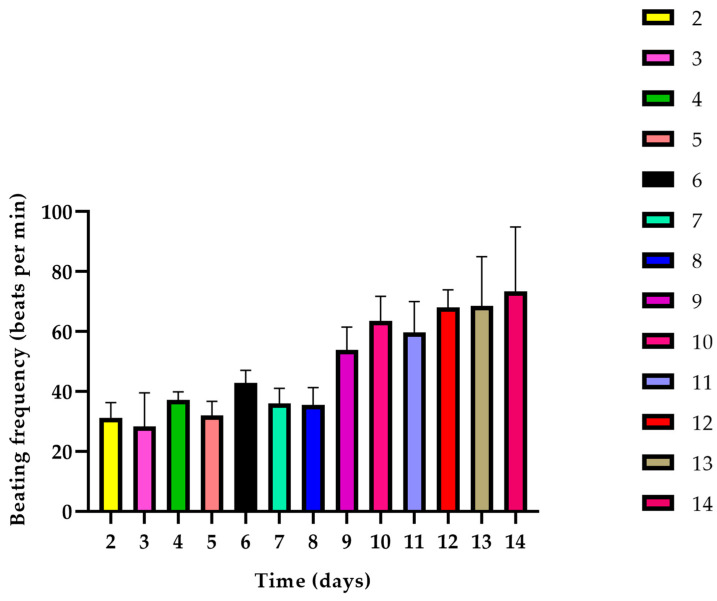
Beating frequencies of the cylindrical fibrin hydrogels embedded with iPSC-CMs, HUVMSCs, and HUVECs from day 2 until day 14.

The beating frequency of the tissue constructs was evaluated from day 2 until day 14 of the cultivation period (Figure 12). The beating frequency values presented an increasing tendency over the cultivation period. However, there were some time intervals observed where the beating frequency value was decreased, i.e., day 3, day 5, day 7, and day 11. However, the beating frequencies of the immediate previous day and the following were not significantly different. Most importantly, on day 3, day 7, and day 10 when the iPSC-CMs containing constructs were inserted in the protective shield, the respective beating frequencies were different from each other with high significance.

## 4. Discussion

In this study, we presented the preparation of DEX-loaded polymeric tubular scaffolds, in conjunction with iPSC-CMs, HUVECs, and HUVSMCs embedded in fibrin gel with potential use as a biological pacemaker. Our evaluation encompassed parameters such as fiber diameter, drug release profiles, cytotoxicity, cell proliferation, and the assessment of construct cell viability through the examination of CD31, αSMA, col I, SAA, and Cx43 expression. Additionally, we measured the beating frequency of the tissue constructs before their integration into the polymeric scaffolds.

Electrospinning yielded polymeric scaffolds with fibrillary structures, featuring nanofiber diameters of 317.38 nm ± 82.57 nm for PLA-based scaffolds and 342.72 nm ± 56.27 nm PLGA-based scaffolds. The topography of electrospun scaffolds was influenced by varying concentrations of loaded DEX, which showed distinct variations for PLA, PLGA, and different DEX concentrations. In particular, the diameter of 40 PLA samples was smaller than that of PLA samples without DEX, aligning with findings from Chen et al. [36] where the diameter of PLA composite fibers increased with higher curcumin concentration. Especially for the PLA concentration that we also used, 15%, the mean diameter of nanofibers belongs to the same range as the fibers that were produced in acetone/dimethylformamide from Casarola et al. [37]. As for the PLGA constructs, the average diameter for our constructs was around 342 nm, which was in the range of a study where PLGA (50:50) [38] or PLGA (75:25) was used [39]. However, in the study of Castaño et al. where 2,2,2-trifluoroethanol was used as a solvent system of 3–9% *w*/*w* PLA, various PLA nanofibers were obtained ranging from 350 to 1300 nm [40].

Regarding the drug release capability of the nanofibrillary scaffolds, after 50 days of incubation, the PLA scaffold loaded with 20 mg/mL DEX reached almost 5%, while the 40 PLA scaffold almost reached 10% (Figure 3g). In contrast, in PCL and PLLA, Vacanti et al. proved the 80% of DEX release at the same incubation time [41], while Tsiapla et al. proved 30% of DEX release in cellulose acetate scaffolds [42], a fact that could be explained from the faster polymer degradation. In another study, the DEX release of a 50% PLA scaffold with a similar molecular weight reached 3% [43]. Although the release values obtained in the present study were similar, our PLA concentration was lower (15%). For the PLGA electrospun scaffolds, unlike the behavior of the PLGA (75:25) in the study of Yoon et al. where a burst release of DEX was observed [44], the DEX release was slower. The PLGA scaffolds loaded with the higher DEX concentration (40 PLGA) performed an initial slower and more gradual release than the 20 mg/mL DEX, as observed in the study of Chen et al. [45]. Moreover, for a 10% PLGA (50:50), the DEX release value was around 2.5% on day 50 [46]. Zheng et al. confirmed that the DEX release can be directly affected by the polymer type, as the PLA-related DEX release value was detected between the different copolymer compositions of the PLGA used [47]. Our study aimed to sustain the initial DEX amount as long as possible in the protective shield and that was feasible with the trifluroethanol/chloroform used as the solvent system. No cytotoxic effect was observed in most of the samples, so at least in view of the results, the manufacturing process of the scaffolds and the solvent system are adequate, and regarding the diameter, there were no significant differences among the samples (Figure S3a). Although in previous studies, it has been seen that the solvent system could influence cell proliferation [48], in this case, the influence of topography on cell proliferation was not observed.

In the current system, the 40 PLA sample achieved the highest drug release on day 50 of the cultivation, while the 20 PLA sample presented a lower release. This result was supported by the fact that the 40 PLA sample fiber diameter values were lower than the 20 PLA sample and considering the higher DEX concentration of the 40 PLA sample, the final drug release value was also higher. As for the PLGA samples, the average 20 PLGA sample fiber diameter was slightly higher than the 40 PLGA fiber. All in all, the co-glucolid content in the PLGA structure is promising a faster degradation than the PLA constructs. However, further research is needed to explore diverse release profiles designed through variations in polymer type, drug concentrations, and solvent systems.

Furthermore, the cytocompatibility tests indicated that the 40 PLGA sample exhibited the highest cytotoxicity for both cell populations tested. In particular, the LDH activity for the 40 PLGA sample was slightly higher for the HUVECs than for the HUVSMCs (Figure 4). Additionally, the fact that the 40 PLGA value was significantly higher for the HUVECs confirmed the importance of DEX concentration in the PLGA polymeric shield. These results are consistent with the cell proliferation results, where the 40 PLGA sample showed the lowest proliferation percentage value among the samples on day 7 of cultivation (Figure 6). These outcomes are consistent with a study where DEX-loaded PLA nanoparticles had negligible effects on the viability of HUVECs and even less on the viability of HUVSMCs with increasing DEX concentration [49]. Particularly for PLGA nanoparticles, HUVEC viability was greatly influenced when DEX was included in the experiment [50]. At the same time, the cell proliferation in our constructs was more favorable for the HUVECs and the HUVEC gel sample than the HUVSMC samples. Moreover, no significant changes were observed among the samples, but cell proliferation in the fibrin gels exhibited a similar trend, which could not be concluded for the HUVEC and HUVSMC samples. To the best of our knowledge, there are no specific studies in the literature that combine polymeric cytotoxicity and proliferation together with DEX, HUVECs, and HUVSMCs. As for the DEX alone, it has been proven to enhance the anti-inflammatory responses of endothelial cells [51], although it did not modify NAD or ATP levels in vivo or in cultured endothelial cells [52]. Additionally, it has been confirmed to upregulate Nox1 expression in vascular smooth muscle cells [53]. This is a reason that could explain our results for the decreased HUVSMC proliferation (Figure S4).

Concerning the CD31 expression in HUVECs and also the αSMA and col I expression in HUVSMCs, the CD31 levels for the PLGA samples were higher than for the PLA samples on day 12 (Figure 7). However, the typical cobblestone structure of the CD31 marker was better imprinted in the PLA samples (Figure S5) than in the PLGA samples (Figure S6) where the DAPI signal was generally comparable between the two types of polymers. Simultaneously, the nuclei appeared more uniform for the PLA samples, especially the PLA sample on day 12 and the 40 PLA sample on day 8. This result was consistent with the study of Shi et al. where PLGA nanoparticles promoted endothelial cell dysfunction [54]. Similarly, in HUVSMCs, the PLA pictures presented a better illustration of the cell nuclei, αSMA, and col I (Figure S8). Indeed, this was in accordance with the quantification analysis of the samples, where the αSMA expression was higher in the PLA samples, unlike the PLGA samples (Figure S9). However, col I levels were changing during the cultivation time points for both polymers, but the final PLA sample on day 12 displayed the most efficient performance (Figure 8). In contrast, a study on coronary smooth muscle cells reported an increase in αSMA and col I expression when PLGA nanoparticles were included in the culture [55].

Taking a closer look at the iPSC-CM samples, a general observation concluded that the PLA sample microscopy evaluations (Figure 9) were more satisfactory than the respective PLGA sample ones (Figure 10). As for the SAA and Cx43 expression levels, they were mostly similar for both polymers, with differences observed on day 7 (20 PLGA, 40 PLGA) and day 10, when the Cx43 values were very low for PLA, 40 PLA, 20 PLGA, and 40 PLGA. In the literature, many studies have used polymeric scaffolds cultivated with iPSC-CMs and have proven satisfactory Cx43 and SAA expression on the PLA/PANI blend [56], PLGA [57], and also PLA and PLGA mixtures with PEG, thus demonstrating that primary cardiomyocytes preferred the hydrophobic PLA and PLGA surface over PEG [58]. Particularly for the iPSC-CMs, DEX has been proven to hinder the premature differentiation in primary cardiomyocytes [59], but in another study, it was shown to stimulate premature terminal differentiation in newborn cardiomyocytes [60]. Another advantageous use of DEX was the successful anti-apoptotic gene carriage with simultaneous low cytotoxicity levels [61]. Moreover, the beating frequency of neonatal cardiac cells was not influenced [62]. However, we cannot correlate that study with our experiments, since we used a higher amount of DEX in our experiments. Nevertheless, the expression of SAA was upregulated in most of the samples that included DEX, except for day 3 of cultivation of the gel in the DEX-embedded samples (Figure 11). However, the DEX concentration affected the Cx43 expression on day 10 of the cultivation in the shield.

## 5. Conclusions

We produced tubular polymeric constructs with the electrospinning method that were used as protective shields for HUVECs, HUVSMCs, and iPSC-CMs embedded in fibrin gels. We characterized the electrospun scaffolds, and we also performed cell cytotoxicity studies for the HUVECs and HUVSMCs showing that the most cytocompatible polymeric sample was the PLA one, while the cell proliferation results showed no significant differences among HUVECs and HUVSMCs. Furthermore, from the CD31, αSMA, col I, SAA, Cx43 imaging, and quantification, we concluded that both polymeric materials can perform as components in biological pacemakers by releasing DEX in different concentrations. Particularly, the PLA samples can be used as presented for an overall more satisfactory performance, while it was proven that DEX has an upregulatory role in maintaining the SAA in the PLGA samples.

## Figures and Tables

**Table 1:** DEX concentration in the PLA/PLGA solutions.

Polymeric Solution	DEX Concentration (mg/mL)
PLA	0
20 PLA	20
40 PLA	40
PLGA	0
20 PLGA	20
40 PLGA	40

**Table 2:** Antibodies for sample staining.

Antibody	Dilution	Supplier
Anti-α-actinin (sarcomeric), mouse monoclonal (primary)	1:50	Sigma-Aldrich (Saint Louis, MO, USA)
Connexin 43 (polyclonal) (primany)	1:50	Thermo Fischer (Waltham, MA, USA)
Connexin 43 monoclonal antibody (3D8A5) (primary)	1:50	Thermo Fischer (Waltham, MA, USA)
Anti-CD31 (PECAM-1) mouse monoclonal (primary)	1:100	Sigma-Aldrich (Saint Louis, MO, USA)
CD31 rabbit polyclonal (primary)	1:100	Abbiotec (Escondido, CA, USA)
α-smooth muscle actin (monoclonal, clone 1A4) (primary)	1:1000	Sigma-Aldrich (Saint Louis, MO, USA)
Anti-collagen I antibody (ab34710) (primary)	1:200	Abcam (Cambridge, UK)
Alexa 594 goat anti-mouse (A11005) (secondary)	1:400	Thermo Fischer (Waltham, MA, USA)
Alexa 594 goat anti-rabbit (A110012) (secondary)	1:400	Thermo Fischer (Waltham, MA, USA)
Alexa 488 goat anti-mouse (A11001) (secondary)	1:400	Thermo Fischer (Waltham, MA, USA)
Alexa 488 goat anti-mouse (A11008) (secondary)	1:400	Thermo Fischer (Waltham, MA, USA)

## Data Availability

Data associated with this study are available upon request to the corresponding author.

## Supplementary Materials

The following supporting information can be downloaded at: https://www.mdpi.com/article/10.3390/polym15214332/s1. Figure S1. Calibration curve for dexamethasone, Figure S2. (a–f) SEM micrographs of the electrospun scaffolds manufactured from PLA and PLGA solutions loaded with varying DEX concentrations in 5000× magnification. (g) Uniform tubular structure of the PLA construct. (h,i) Cross-sectional cuts of the PLA construct, Figure S3. (a) Fiber diameter for the PLA, 20 PLA, 40 PLA, PLGA, 20 PLGA and 40 PLGA constructs. (b) Average fiber diameter chart of the PLA and the PLGA constructs, Figure S4. Cell proliferation of the HUVECs, HUVSMCs, HUVEC gel and HUVSCM gel for the PLA and PLGA samples on day 1 (top left), day 3 (top right) and day 7 (bottom). Data are expressed as mean ± SD, *n* = 3, ** *p* < 0.01, *** *p* < 0.001, **** *p* < 0.0001), Figure S5. DAPI (blue) and CD31 (red) staining for the PLA (left), 20 PLA (middle) and 40 PLA samples (right) on day 4 (first row), day 8 (second row) and day 12 (third row), Figure S6. DAPI (blue) and CD31 (red) staining for the PLGA (left), 20 PLGA (middle) and 40 PLGA samples (right) on day 4 (first row), day 8 (second row) and day 12 (third row), Figure S7. Quantification of DAPI and CD31 for the PLA (a) and PLGA (b) samples (Data are expressed as mean ± SD, *n* = 3, * *p* < 0.05, ** *p* < 0.01, **** *p* < 0.0001), Figure S8. DAPI (blue), αSMA (red) and col I (green) staining for the PLA (left), 20 PLA (middle) and 40 PLA samples (right) on day 4 (first row), day 8 (second row) and day 12 (third row), Figure S9. DAPI (blue), αSMA (red) and col I (green) staining for the PLGA (left), 20 PLGA (middle) and 40 PLGA samples (right) on day 4 (first row), day 8 (second row) and day 12 (third row), Figure S10. Quantification of DAPI, col I and αSMA for the PLA (a) and PLGA (b) samples (Data is expressed as mean ± SD, *n* = 3, * *p* < 0.05, ** *p* < 0.01, **** *p* < 0.0001).

## References

[1] Gaziano T., Reddy K.S., Paccaud F., Horton S., Chaturvedi V. (2006). Cardiovascular Disease. Disease Control Priorities in Developing Countries.

[2] Nugent H.M., Edelman E.R. (2003). Tissue engineering therapy for cardiovascular disease. Circ. Res..

[3] Zoob M., Smith K.S. (1963). Aetiology of complete heart-block. Br. Med. J..

[4] Pyngottu A., Werner H., Lehmann P., Balmer C. (2019). Health-related quality of life and psychological adjustment of children and adolescents with pacemakers and implantable cardioverter defibrillators: A systematic review. Pediatr. Cardiol..

[5] Bernstein A.D., Parsonnet V. (1996). Survey of cardiac pacing and defibrillation in the United States in 1993. Am. J. Cardiol..

[6] Kiehl E.L., Makki T., Matar R.M., Johnston D.R., Rickard J.W., Tarakji K.G., Kanj M., Wazni O.M., Saliba W.I., Varma N. (2017). Incidence and predictors of late atrioventricular conduction recovery among patients requiring permanent pacemaker for complete heart block after cardiac surgery. Heart Rhythm.

[7] Liberman L., Silver E.S., Chai P.J., Anderson B.R. (2016). Incidence and characteristics of heart block after heart surgery in pediatric patients: A multicenter study. J. Thorac. Cardiovasc. Surg..

[8] Takeuchi D., Tomizawa Y. (2013). Pacing device therapy in infants and children: A review. J. Artif. Organs.

[9] Ronaldson-Bouchard K., Ma S.P., Yeager K., Chen T., Song L., Sirabella D., Morikawa K., Teles D., Yazawa M., Vunjak-Novakovic G. (2018). Advanced maturation of human cardiac tissue grown from pluripotent stem cells. Nature.

[10] Campostrini G., Windt L.M., van Meer B.J., Bellin M., Mummery C.L. (2021). Cardiac tissues from stem cells: New routes to maturation and cardiac regeneration. Circ. Res..

[11] Chauveau S., Anyukhovsky E.P., Ben-Ari M., Naor S., Jiang Y.-P., Danilo P., Rahim T., Burke S., Qiu X., Potapova I.A. (2017). Induced pluripotent stem cell–derived cardiomyocytes provide in vivo biological pacemaker function. Circulation Arrhythmia Electrophysiol..

[12] Zhang W., Li X., Sun S., Zhang X. (2019). Implantation of engineered conduction tissue in the rat heart. Mol. Med. Rep..

[13] Murata K., Masumoto H. (2022). Systems for the functional evaluation of human heart tissues derived from pluripotent stem cells. Stem Cells.

[14] Miller J.S., Stevens K.R., Yang M.T., Baker B.M., Nguyen D.-H.T., Cohen D.M., Toro E., Chen A.A., Galie P.A., Yu X. (2012). Rapid casting of patterned vascular networks for perfusable engineered three-dimensional tissues. Nat. Mater..

[15] Brassard J.A., Nikolaev M., Hübscher T., Hofer M., Lutolf M.P. (2021). Recapitulating macro-scale tissue self-organization through organoid bioprinting. Nat. Mater..

[16] Skylar-Scott M.A., Uzel S.G., Nam L.L., Ahrens J.H., Truby R.L., Damaraju S., Lewis J.A. (2019). Biomanufacturing of organ-specific tissues with high cellular density and embedded vascular channels. Sci. Adv..

[17] Cairns M.L., Dickson G.R., Orr J.F., Farrar D., Hardacre C., Sa J., Lemoine P., Mughal M.Z., Buchanan F.J. (2012). The potential of electron beam radiation for simultaneous surface modification and bioresorption control of PLLA. J. Biomed. Mater. Res. Part A.

[18] Li P., Feng X., Jia X., Fan Y. (2010). Influences of tensile load on in vitro degradation of an electrospun poly (l-lactide-co-glycolide) scaffold. Acta Biomater..

[19] Jun I., Han H.-S., Edwards J.R., Jeon H. (2018). Electrospun fibrous scaffolds for tissue engineering: Viewpoints on architecture and fabrication. Int. J. Mol. Sci..

[20] Joanne P., Kitsara M., Boitard S.-E., Naemetalla H., Vanneaux V., Pernot M., Larghero J., Forest P., Chen Y., Menasché P. (2016). Nanofibrous clinical-grade collagen scaffolds seeded with human cardiomyocytes induces cardiac remodeling in dilated cardiomyopathy. Biomaterials.

[21] Li J., Minami I., Yu L., Tsuji K., Nakajima M., Qiao J., Suzuki M., Shimono K., Nakatsuji N., Kotera H. (2016). Extracellular recordings of patterned human pluripotent stem cell-derived cardiomyocytes on aligned fibers. Stem Cells Int..

[22] Wanjare M., Hou L., Nakayama K.H., Kim J.J., Mezak N.P., Abilez O.J., Tzatzalos E., Wu J.C., Huang N.F. (2017). Anisotropic microfibrous scaffolds enhance the organization and function of cardiomyocytes derived from induced pluripotent stem cells. Biomater. Sci..

[23] Han J., Wu Q., Xia Y., Wagner M.B., Xu C. (2016). Cell alignment induced by anisotropic electrospun fibrous scaffolds alone has limited effect on cardiomyocyte maturation. Stem Cell Res..

[24] Chun Y.W., Balikov D.A., Feaster T.K., Williams C.H., Sheng C.C., Lee J.-B., Boire T.C., Neely M.D., Bellan L.M., Ess K.C. (2015). Combinatorial polymer matrices enhance in vitro maturation of human induced pluripotent stem cell-derived cardiomyocytes. Biomaterials.

[25] Xu C., Miranda-Nieves D., Ankrum J.A., Matthiesen M.E., Phillips J.A., Roes I., Wojtkiewicz G.R., Juneja V., Kultima J.R., Zhao W. (2012). Tracking mesenchymal stem cells with iron oxide nanoparticle loaded poly (lactide-co-glycolide) microparticles. Nano Lett..

[26] Ankrum J.A., Dastidar R.G., Ong J.F., Levy O., Karp J.M. (2014). Performance-enhanced mesenchymal stem cells via intracellular delivery of steroids. Sci. Rep..

[27] Johnson D.B., Lopez M.J., Kelley B. (2018). Dexamethasone. https://pubmed.ncbi.nlm.nih.gov/29489240/.

[28] Rog-Zielinska E.A., Richardson R.V., Denvir M.A., Chapman K.E. (2014). Glucocorticoids and foetal heart maturation; implications for prematurity and foetal programming. J. Mol. Endocrinol..

[29] Parikh S.S., Blackwell D.J., Gomez-Hurtado N., Frisk M., Wang L., Kim K., Dahl C.P., Fiane A., Tønnessen T., Kryshtal D.O. (2017). Thyroid and glucocorticoid hormones promote functional T-tubule development in human-induced pluripotent stem cell–derived cardiomyocytes. Circ. Res..

[30] El Zaoui I., Behar-Cohen F., Torriglia A. (2015). Glucocorticoids exert direct toxicity on microvasculature: Analysis of cell death mechanisms. Toxicol. Sci..

[31] Doshi J., Reneker D.H. (1995). Electrospinning process and applications of electrospun fibers. J. Electrost..

[32] Hotaling N.A., Bharti K., Kriel H., Simon C.G. (2015). DiameterJ: A validated open source nanofiber diameter measurement tool. Biomaterials.

[33] Keijdener H., Konrad J., Hoffmann B., Gerardo-Nava J., Rütten S., Merkel R., Vázquez-Jiménez J., Brook G.A., Jockenhoevel S., Mela P. (2020). A bench-top molding method for the production of cell-laden fibrin micro-fibers with longitudinal topography. J. Biomed. Mater. Res. Part B Appl. Biomater..

[34] Kyriakou S., Lubig A., Sandhoff C.A., Kuhn Y., Jockenhoevel S. (2023). Influence of Diameter and Cyclic Mechanical Stimulation on the Beating Frequency of Myocardial Cell-Laden Fibers. Gels.

[35] Moreira R., Velz T., Alves N., Gesche V.N., Malischewski A., Schmitz-Rode T., Frese J., Jockenhoevel S., Mela P. (2015). Tissue-engineered heart valve with a tubular leaflet design for minimally invasive transcatheter implantation. Tissue Eng. Part C Methods.

[36] Chen Y., Lin J., Fei Y., Wang H., Gao W. (2010). Preparation and characterization of electrospinning PLA/curcumin composite membranes. Fibers Polym..

[37] Casasola R., Thomas N.L., Trybala A., Georgiadou S. (2014). Electrospun poly lactic acid (PLA) fibres: Effect of different solvent systems on fibre morphology and diameter. Polymer.

[38] Zhao L., He C., Gao Y., Cen L., Cui L., Cao Y. (2008). Preparation and cytocompatibility of PLGA scaffolds with controllable fiber morphology and diameter using electrospinning method. J. Biomed. Mater. Res. Part B Appl. Biomater..

[39] Liu X., Baldursdottir S.G., Aho J., Qu H., Christensen L.P., Rantanen J., Yang M. (2017). Electrospinnability of poly lactic-co-glycolic acid (PLGA): The role of solvent type and solvent composition. Pharm. Res..

[40] Castaño Linares Ó., López Mengual A., Reginensi D., Matamoros Angles A., Engel López E., del Río Fernández J.A. (2021). Chemotactic TEG3 cells’ guiding platforms based on PLA fibers functionalized with the SDF-1a/CXCL12 chemokine for neural regeneration therapy. Front. Bioeng. Biotechnol..

[41] Vacanti N.M., Cheng H., Hill P.S., Guerreiro J.O.D., Dang T.T., Ma M., Watson S.E., Hwang N.S., Langer R., Anderson D.G. (2012). Localized delivery of dexamethasone from electrospun fibers reduces the foreign body response. Biomacromolecules.

[42] Tsiapla A.-R., Karagkiozaki V., Bakola V., Pappa F., Gkertsiou P., Pavlidou E., Logothetidis S. (2018). Biomimetic and biodegradable cellulose acetate scaffolds loaded with dexamethasone for bone implants. Beilstein J. Nanotechnol..

[43] Li D., Guo G., Fan R., Liang J., Deng X., Luo F., Qian Z. (2013). PLA/F68/Dexamethasone implants prepared by hot-melt extrusion for controlled release of anti-inflammatory drug to implantable medical devices: I. Preparation, characterization and hydrolytic degradation study. Int. J. Pharm..

[44] Yoon J.J., Kim J.H., Park T.G. (2003). Dexamethasone-releasing biodegradable polymer scaffolds fabricated by a gas-foaming/salt-leaching method. Biomaterials.

[45] Chen D., Luo Y., Pan J., Chen A., Ma D., Xu M., Tang J., Zhang H. (2021). Long-term release of dexamethasone with a polycaprolactone-coated electrode alleviates fibrosis in cochlear implantation. Front. Cell Dev. Biol..

[46] Barbosa-Alfaro D., Andres-Guerrero V., Fernandez-Bueno I., García-Gutiérrez M.T., Gil-Alegre E., Molina-Martínez I.T., Pastor-Jimeno J.C., Herrero-Vanrell R., Bravo-Osuna I. (2021). Dexamethasone plga microspheres for sub-tenon administration: Influence of sterilization and tolerance studies. Pharmaceutics.

[47] Zheng A., Waterkotte T., Debele T., Dion G., Park Y. (2023). Biodegradable dexamethasone polymer capsule for long-term release. Korean J. Chem. Eng..

[48] Gu Z., Fan S., Kundu S.C., Yao X., Zhang Y. (2023). Fiber diameters and parallel patterns: Proliferation and osteogenesis of stem cells. Regen. Biomater..

[49] Xu L., Wang H., Tian H., Zhang M., He J., Ni P. (2021). Facile construction of noncovalent graft copolymers with triple stimuli-responsiveness for triggered drug delivery. Polym. Chem..

[50] Li Z., Huang H., Huang L., Du L., Sun Y., Duan Y. (2017). Prevention of oxidized low density lipoprotein-induced endothelial cell injury by DA-PLGA-PEG-cRGD nanoparticles combined with ultrasound. Int. J. Mol. Sci..

[51] Ding Y., Gao Z.-G., Jacobson K.A., Suffredini A.F. (2010). Dexamethasone enhances ATP-induced inflammatory responses in endothelial cells. J. Pharmacol. Exp. Ther..

[52] Herrera N.A., Duchatsch F., Kahlke A., Amaral S.L., Vasquez-Vivar J. (2020). In vivo vascular rarefaction and hypertension induced by dexamethasone are related to phosphatase PTP1B activation not endothelial metabolic changes. Free. Radic. Biol. Med..

[53] Siuda D., Tobias S., Rus A., Xia N., Förstermann U., Li H. (2014). Dexamethasone upregulates Nox1 expression in vascular smooth muscle cells. Pharmacology.

[54] Shi W., Fuad A.R.M., Li Y., Wang Y., Huang J., Du R., Wang G., Wang Y., Yin T. (2023). Biodegradable polymeric nanoparticles increase risk of cardiovascular diseases by inducing endothelium dysfunction and inflammation. J. Nanobiotechnology.

[55] Rezaei H., Rezaie Z., Seifati S.M., Ardeshirylajimi A., Basiri A., Taheri M., Omrani M.D. (2020). Poly-phosphate increases SMC differentiation of mesenchymal stem cells on PLGA–polyurethane nanofibrous scaffold. Cell Tissue Bank..

[56] Khan M., Xu Y., Hua S., Johnson J., Belevych A., Janssen P.M., Gyorke S., Guan J., Angelos M.G. (2015). Evaluation of changes in morphology and function of human induced pluripotent stem cell derived cardiomyocytes (HiPSC-CMs) cultured on an aligned-nanofiber cardiac patch. PLoS ONE.

[57] Hsiao C.-W., Bai M.-Y., Chang Y., Chung M.-F., Lee T.-Y., Wu C.-T., Maiti B., Liao Z.-X., Li R.-K., Sung H.-W. (2013). Electrical coupling of isolated cardiomyocyte clusters grown on aligned conductive nanofibrous meshes for their synchronized beating. Biomaterials.

[58] Zong X., Bien H., Chung C.-Y., Yin L., Fang D., Hsiao B.S., Chu B., Entcheva E. (2005). Electrospun fine-textured scaffolds for heart tissue constructs. Biomaterials.

[59] Gay M.S., Li Y., Xiong F., Lin T., Zhang L. (2015). Dexamethasone treatment of newborn rats decreases cardiomyocyte endowment in the developing heart through epigenetic modifications. PLoS ONE.

[60] Gay M.S., Dasgupta C., Li Y., Kanna A., Zhang L. (2016). Dexamethasone induces cardiomyocyte terminal differentiation via epigenetic repression of cyclin D2 gene. J. Pharmacol. Exp. Ther..

[61] Kim H., Kim H.A., Bae Y.M., Choi J.S., Lee M. (2009). Dexamethasone-conjugated polyethylenimine as an efficient gene carrier with an anti-apoptotic effect to cardiomyocytes. J. Gene Med..

[62] Rossier M.F., Lenglet S.B., Vetterli L., Python M., Maturana A.S. (2008). Corticosteroids and redox potential modulate spontaneous contractions in isolated rat ventricular cardiomyocytes. Hypertension.

