# Supplementary material for: A Dexamethasone-Loaded Polymeric Electrospun Construct as a Tubular Cardiovascular Implant
Source: Polymers (Basel). 2023 Nov 6;15(21):4332. doi: 10.3390/polym15214332 (PMC10649717; doi:10.3390/polym15214332)
Supplement: Supplementary file 1 [file polymers-15-04332-s001.zip › polymers-2683551-supplementary.pdf]

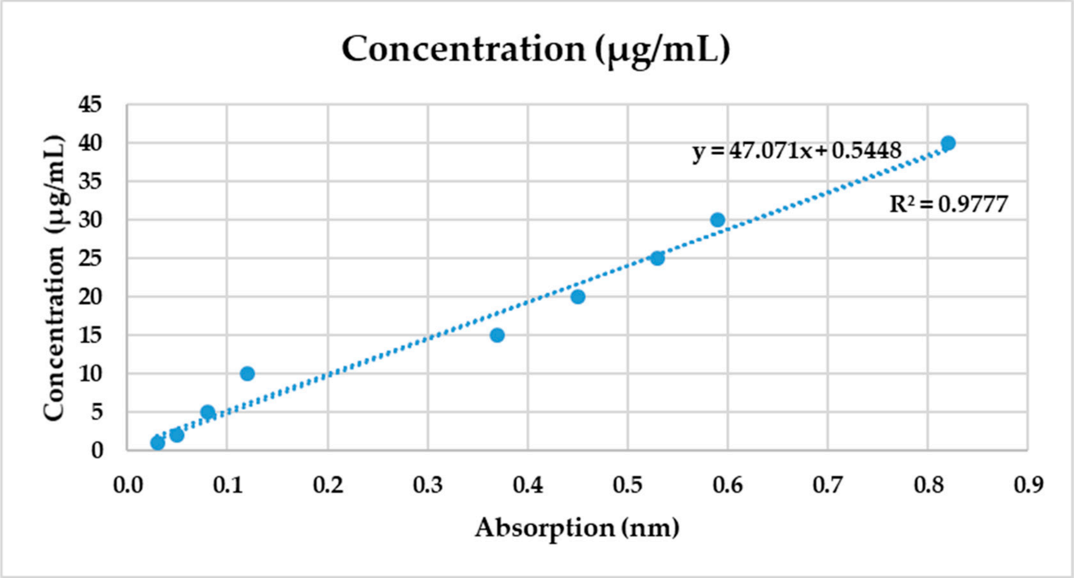

Supplementary Figure S1. Calibration curve for dexamethasone

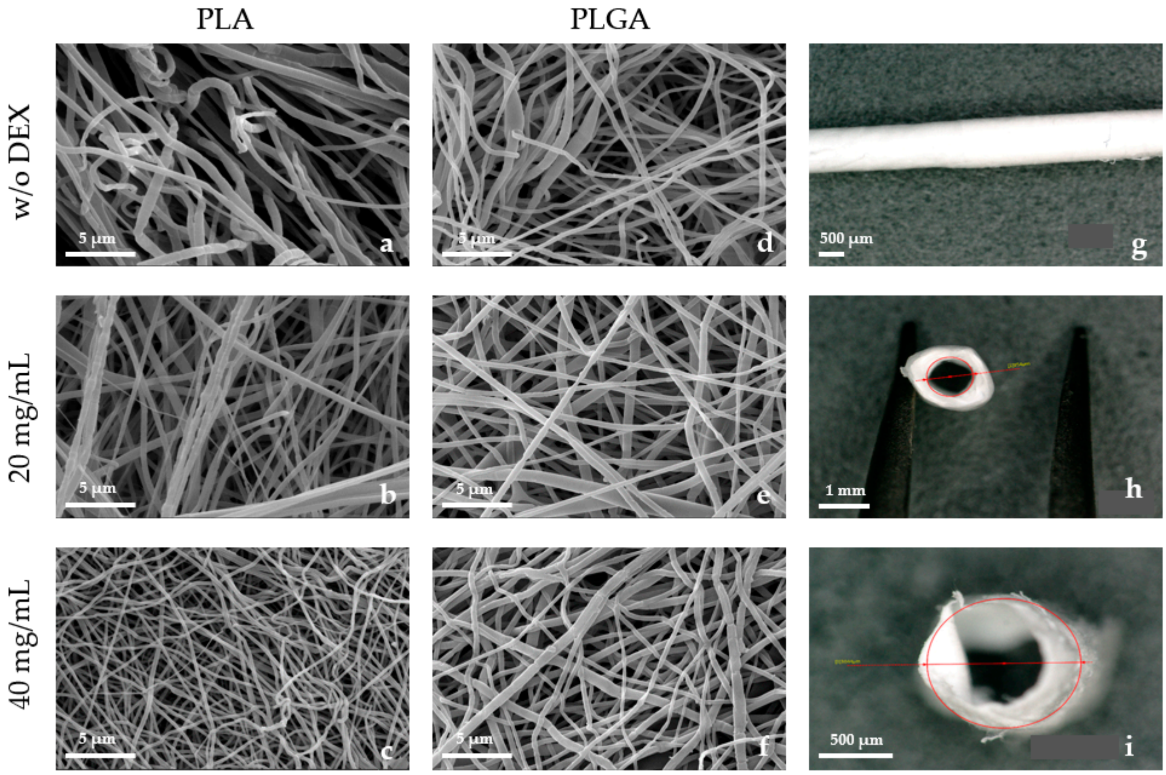

Supplementary Figure S2. (a-f) SEM micrographs of the electrospun scaffolds manufactured from PLA and PLGA solutions loaded with varying DEX concentration in 5000x magnification. (g) Uniform tubular structure of the PLA construct. (h-i) Cross-sectional cuts of the PLA construct.

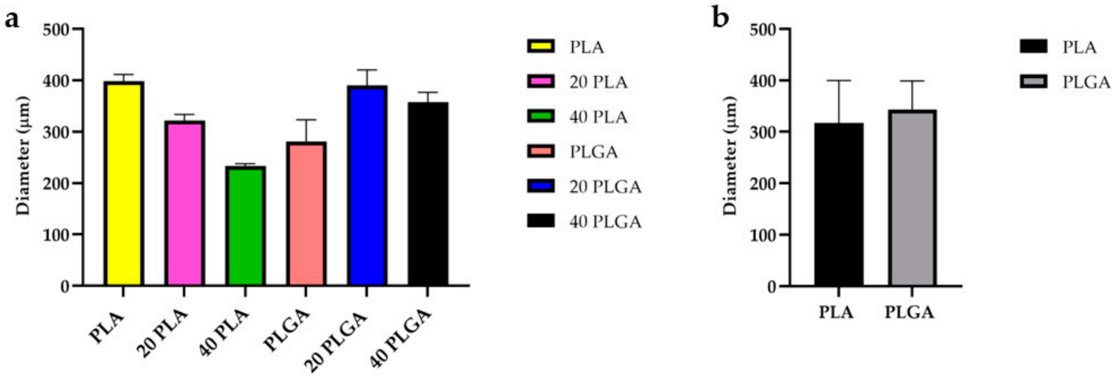

Supplementary Figure S3. (a) Fiber diameter for the PLA, 20 PLA, 40 PLA, PLGA, 20 PLGA and 40 PLGA constructs. (b) Average fiber diameter chart of the PLA and the PLGA constructs.

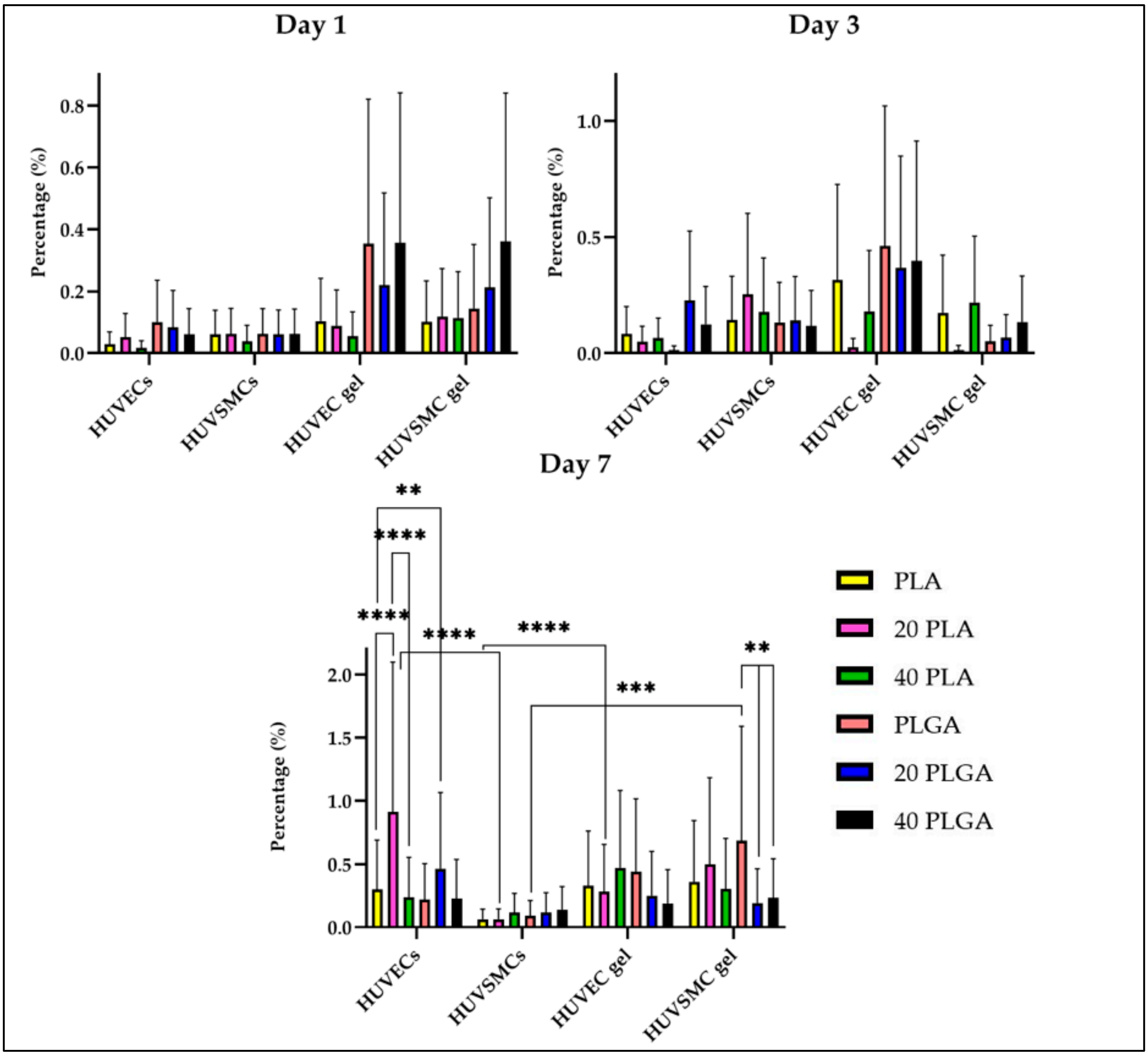

Supplementary Figure S4. Cell proliferation of the HUVECs, HUVSMCs, HUVEC gel and HUVSCM gel for the PLA and PLGA samples on day 1 (top left), day 3 (top right) and day 7 (bottom). Data are expressed as mean ± SD, $n = 3$, $^{**}p < 0.01$, $^{***}p < 0.001$, $^{****}p < 0.0001$).

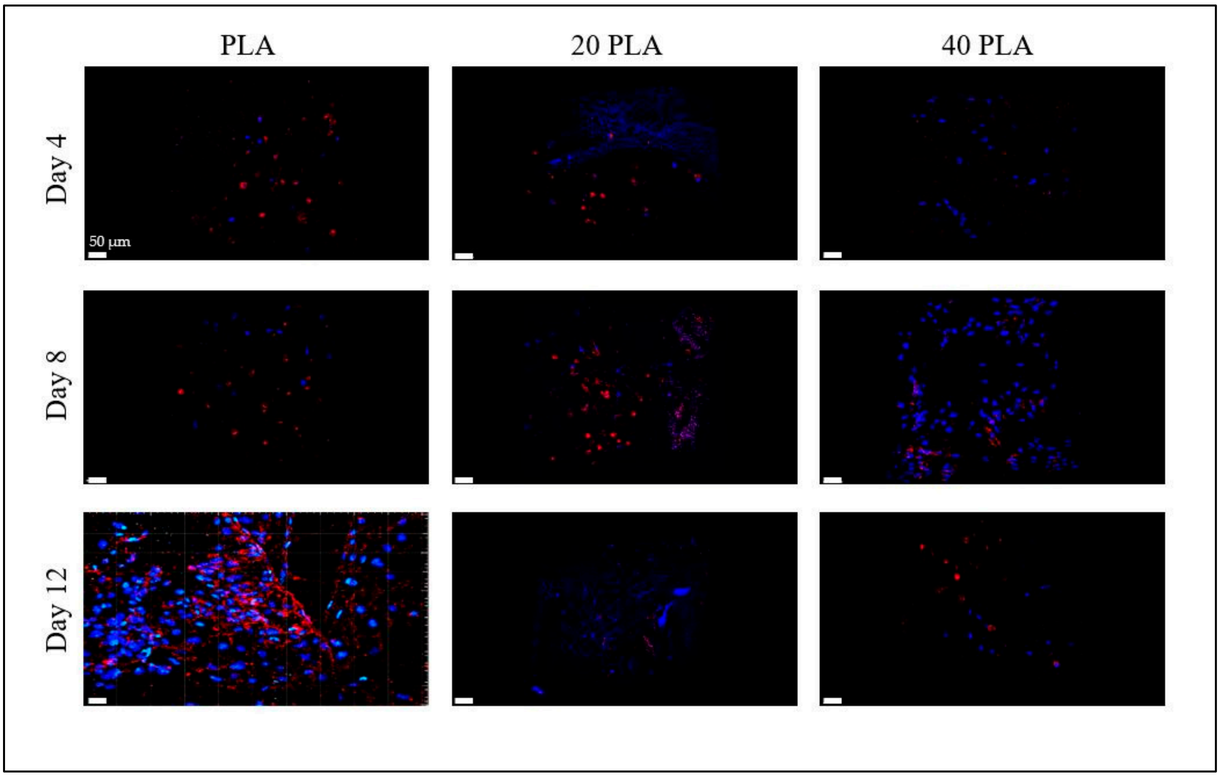

Supplementary Figure S5. DAPI (blue) and CD31 (red) staining for the PLA (left), 20 PLA (middle) and 40 PLA samples (right) on day 4 (first row), day 8 (second row) and day 12 (third row).

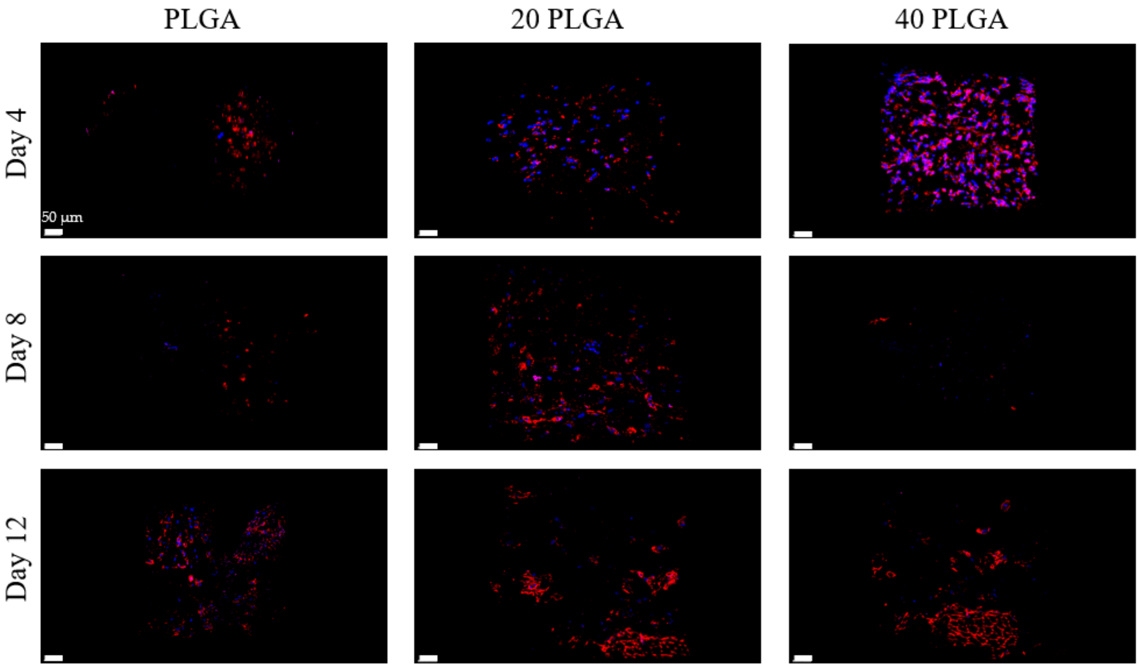

Supplementary Figure S6. DAPI (blue) and CD31 (red) staining for the PLGA (left), 20 PLGA (middle) and 40 PLGA samples (right) on day 4 (first row), day 8 (second row) and day 12 (third row).

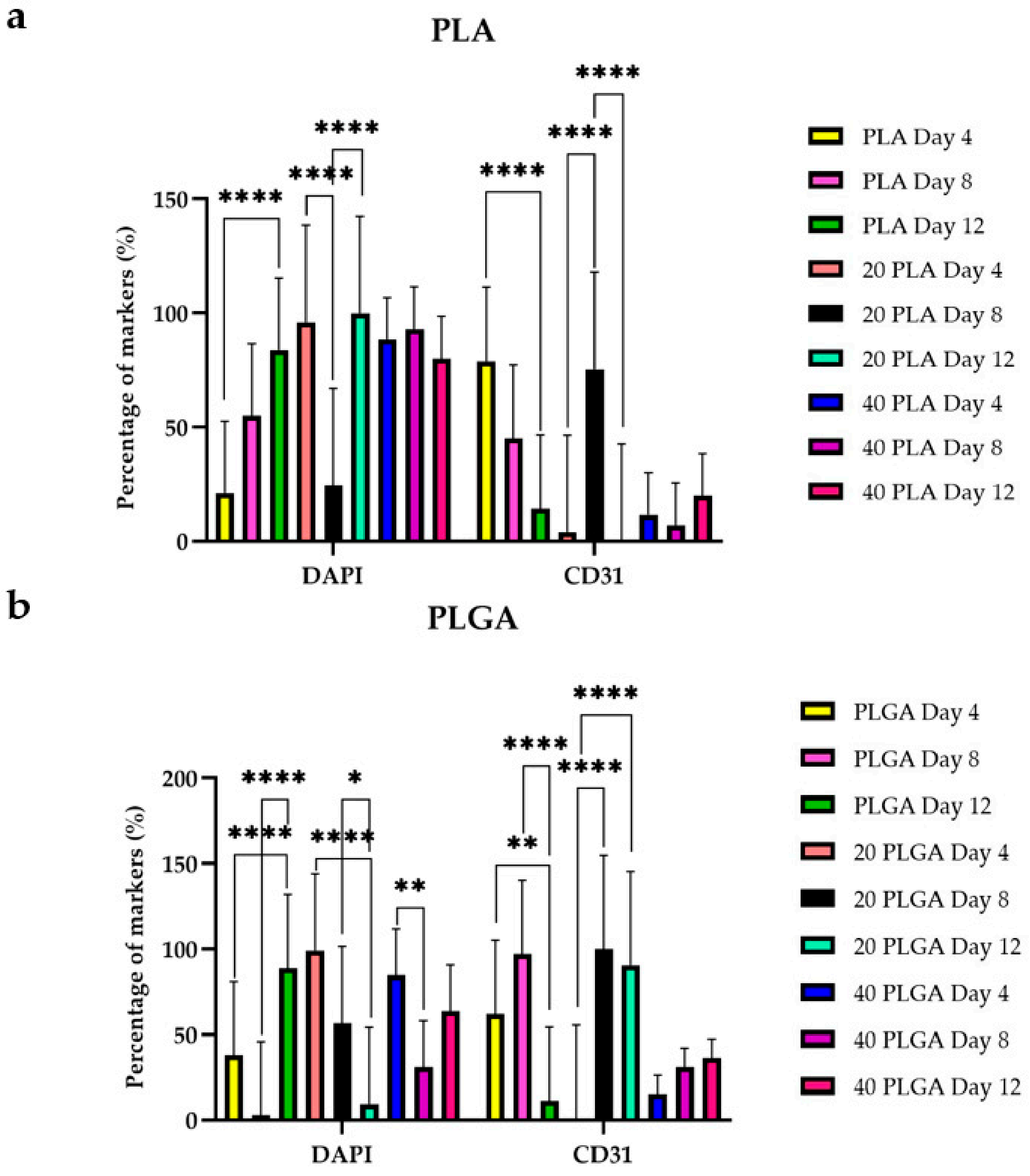

Supplementary Figure S7. Quantification of DAPI and CD31 for the PLA (a) and PLGA (b) samples (Data are expressed as mean ± SD, $n = 3$, *$p < 0.05$, **$p < 0.01$, ****$p < 0.0001$).

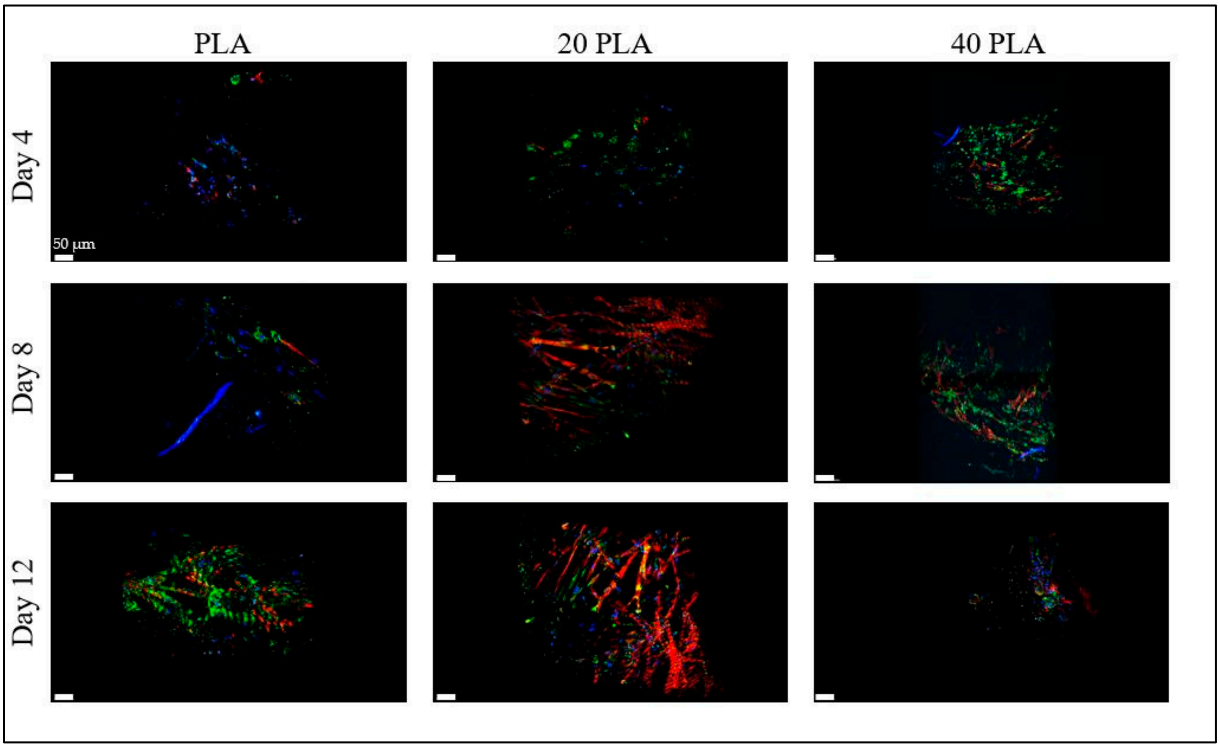

Supplementary Figure S8. DAPI (blue), αSMA (red) and col I (green) staining for the PLA (left), 20 PLA (middle) and 40 PLA samples (right) on day 4 (first row), day 8 (second row) and day 12 (third row).

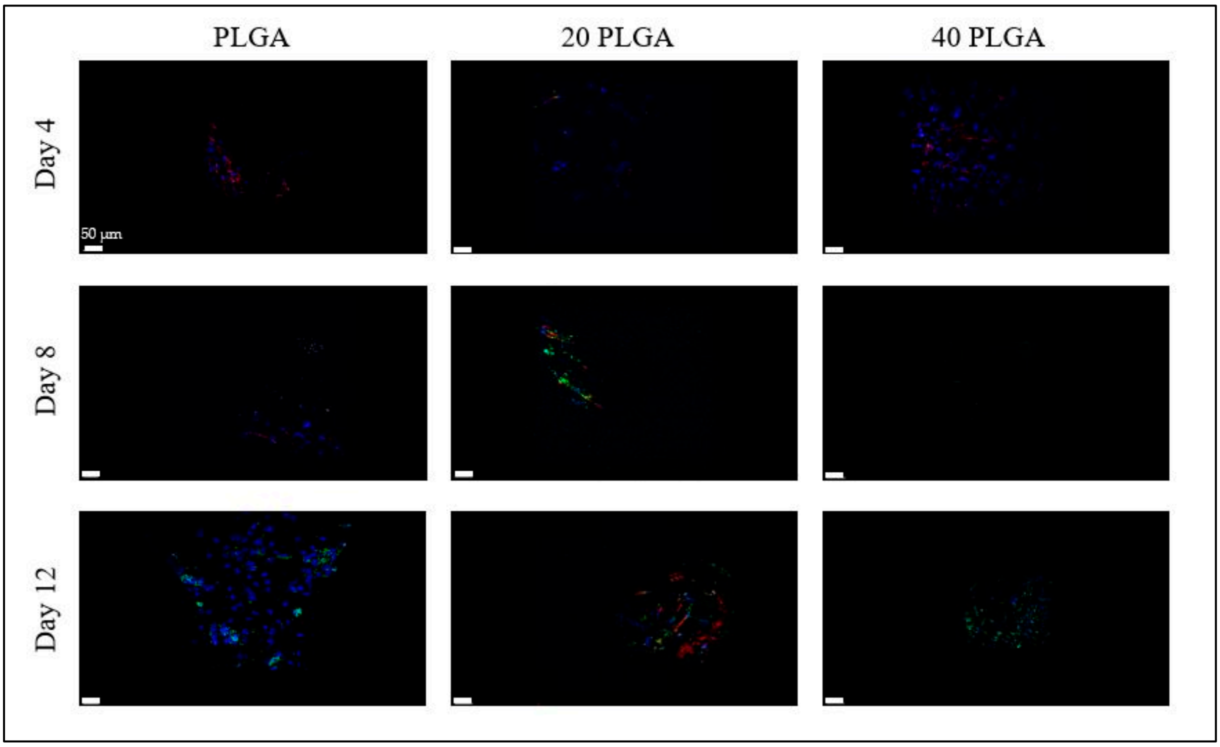

Supplementary Figure S9. DAPI (blue), αSMA (red) and col I (green) staining for the PLGA (left), 20 PLGA (middle) and 40 PLGA samples (right) on day 4 (first row), day 8 (second row) and day 12 (third row).

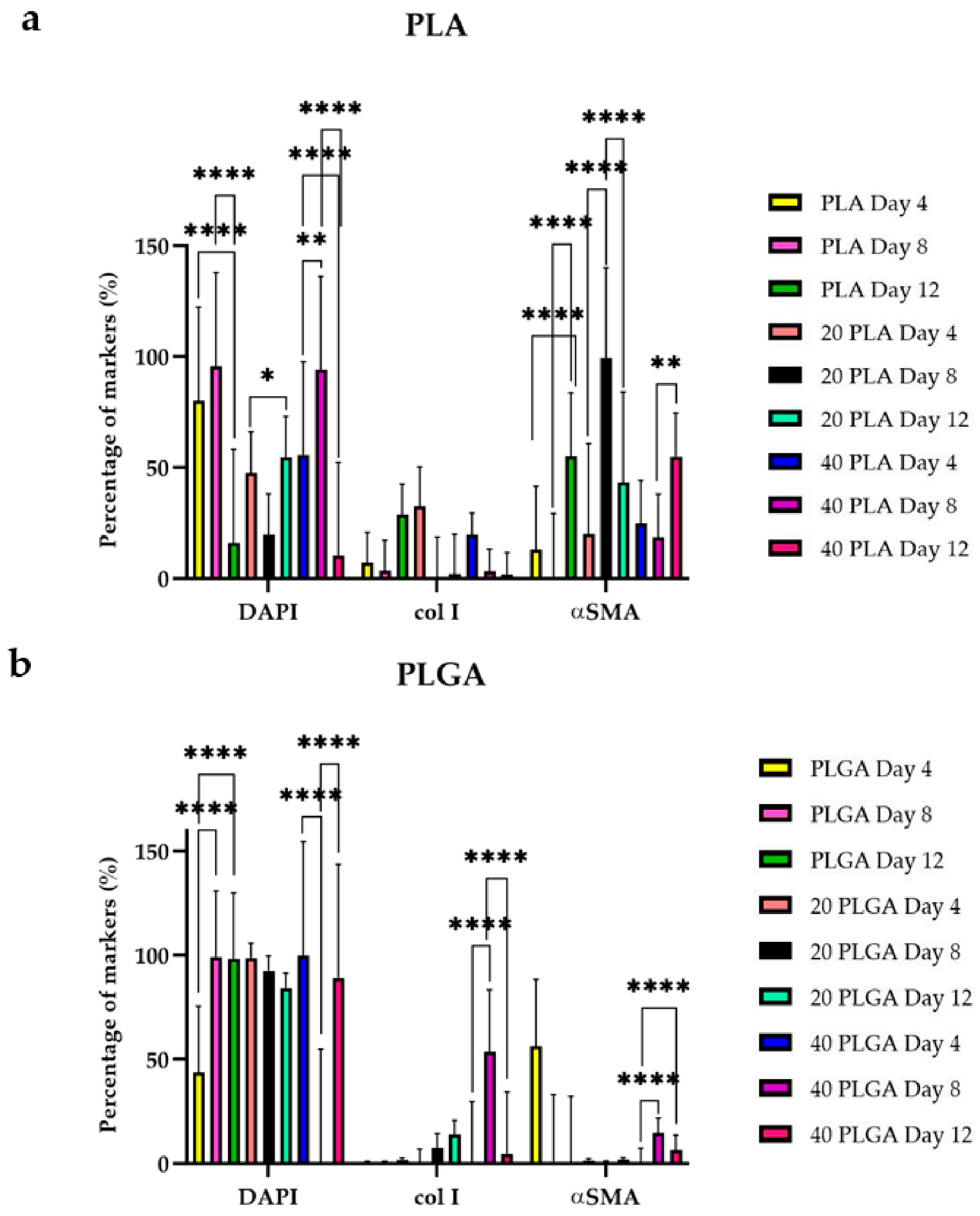

Supplementary Figure S10. Quantification of DAPI, col I and αSMA for the PLA (a) and PLGA (b) samples (Data is expressed as mean ± SD, $n = 3$, $^{*}p < 0.05$, $^{**}p < 0.01$, $^{****}p < 0.0001$).
